# Promise and pitfalls of AI chatbots in complex decision-making for thyroid nodules and papillary thyroid cancer

**DOI:** 10.1530/ETJ-25-0385

**Published:** 2026-04-08

**Authors:** Grigoris Effraimidis, Athanasios Kasotas, Sofia Varsami, Eleni Sazakli, Olga Karapanou, Katerina Saltiki, Marina Michalaki

**Affiliations:** ^1^ Faculty of Medicine, School of Health Sciences, University of Thessaly, Larissa, Greece; ^2^ Department of Endocrinology and Metabolic Diseases, Larissa University Hospital, Larissa, Greece; ^3^ Bioinformatics, Faculty of Science, University of Copenhagen, Copenhagen, Denmark; ^4^ Faculty of Medicine, School of Health Science, University of Patras, Patras, Greece; ^5^ Endocrine Department, NIMTS Veteran’s Hospital, Athens, Greece; ^6^ Endocrine Unit, Department of Clinical Therapeutics, National and Kapodistrian University, Athens, Greece

**Keywords:** artificial intelligence, chatbots, thyroid nodules, papillary thyroid cancer, clinical decision-making, survey

## Abstract

**Introduction:**

Artificial intelligence (AI) chatbots are increasingly used in medicine, but their reliability in scenarios with multiple management options is unclear. Indeterminate thyroid nodules and low- and low-to-intermediate-risk papillary thyroid carcinoma (PTC) represent such cases.

**Methods:**

In a nationwide web-based survey, 201 members of the Hellenic Endocrine Society evaluated 12 clinical vignettes on indeterminate thyroid nodules and low- and low-to-intermediate-risk PTC. Their responses were compared with those generated by four conversational AI models (ChatGPT, Gemini, Copilot, and DeepSeek) at two time points, 11 months apart. DeepSeek was assessed only at the second time point. Chatbot outputs were assessed for agreement with endocrinologists’ predominant answers, concordance with the most guideline-consistent options (American and European Thyroid Association recommendations), temporal stability, and inter-model agreement.

**Results:**

Alignment between chatbots and endocrinologists’ predominant responses was limited, reaching at most 25% across scenarios. In contrast, concordance with the most guideline-consistent options was higher, up to 83% (10/12 scenarios), depending on the model and time point. Across 12 scenarios, ChatGPT, Gemini, and Copilot changed their responses in 4, 7, and 5 scenarios, respectively, with some updates moving closer to, and others further from, guideline-based answers. Inter-model agreement ranged from 33 to 67%, indicating substantial variability among chatbots.

**Conclusion:**

AI chatbots show evolving but inconsistent performance in complex thyroid management scenarios. While guideline concordance can be relatively high, substantial variability across models, limited temporal reproducibility, and poor alignment with clinical practice highlight the need for ongoing longitudinal evaluation before safe integration into clinical decision-making.

## Introduction

Recent advances in artificial intelligence (AI) have led to a paradigmatic shift, with AI-powered technologies increasingly integrated into various fields (1). Τhe adoption of large language models (LLMs), such as AI-driven chatbots, has intrigued researchers by offering novel opportunities in healthcare and medicine. In the field of medicine, AI chatbots have drawn substantial attention due to their potential to complement or enhance medical decision-making, with studies evaluating their ability to assist in diagnosis, treatment recommendations, and medical education. Notably, studies comparing AI-generated responses with those of medical professionals suggest that AI may be preferable in terms of both quality and empathy (2, 3).

The widespread use of high-frequency ultrasonography has led to increased detection of thyroid nodules (4, 5), paralleling a rise in papillary thyroid carcinoma (PTC) diagnoses (6). Although this trend began to reverse after 2015, the overall incidence remains high (7, 8). Given the indolent course of PTC, concerns about overdiagnosis and overtreatment have led to de-escalation strategies, such as less extensive surgery, selective use of radioactive iodine (RAI), and active surveillance (9, 10). As thyroid nodules and thyroid cancer have become increasingly prevalent, patients often seek answers about diagnosis, treatment, and follow-up through online resources (11). With the development of AI technologies, a growing perception that common clinical questions, including those from patients with thyroid nodules or cancer, can be efficiently addressed by AI-driven chatbots makes them easily accessible tools for medical guidance (12, 13).

Clinical scenarios involving indeterminate thyroid nodules and low- to low-to-intermediate-risk PTC are examples of ongoing debates within thyroidology. Although guidelines from the American and European Thyroid Associations (ATA and ETA) (10, 14, 15, 16) provide management frameworks, they often permit multiple acceptable options, leaving room for varied interpretations. Such uncertainty makes these scenarios ideal for evaluating whether AI chatbots provide consistent, guideline-concordant advice. Moreover, since AI models are periodically updated, their outputs may evolve over time, potentially improving or deviating from established medical standards. Evaluating how chatbot recommendations change over time in such debated scenarios is essential to assess their potential utility in clinical decision-making.

Despite their growing popularity, concerns persist regarding the accuracy, consistency, and completeness of the information AI chatbots provide, especially in clinical scenarios (17, 18), creating important questions to be answered: do AI chatbots provide stable evidence-based responses over time, or do their outputs evolve to better align with medical standards and expert opinions as they are updated? Therefore, understanding how AI chatbot responses change over time is essential for evaluating their reliability and defining their role in the future of thyroid nodules and PTC.

To address these issues, we designed a study comparing four conversational AI models’ responses with endocrinologists’ responses in a nationwide web survey on thyroid nodules with indeterminate cytology and low- and low-to-intermediate-risk PTC management across 12 clinical scenarios. Responses were evaluated for concordance with the most guideline-consistent answer based on the ATA and ETA recommendations available at the time of the survey (14, 15, 16). Moreover, AI chatbot responses were evaluated at two points, 11 months apart, to examine their ability to incorporate new information and adjust accordingly over time. Thus, we aim to better understand their reliability and evolution in clinical decision-making. Notably, the study does not seek to determine which AI chatbot performs best.

## Materials and methods

### Study design

From November 2023 to April 2024, a web survey was conducted among members of the Hellenic Endocrine Society (HES) on the management of thyroid nodules with atypia of undetermined significance (AUS) cytology and low-risk and low-to-intermediate-risk PTC, as previously reported (19, 20). The survey was built using SurveyLegend and emailed to all 809 HES members. Participation was voluntary, was anonymous, and aimed to capture at least 20% of the membership. Greek endocrinologists evaluated 12 clinical scenarios, selecting one of three or four management options. The survey also collected demographic data, confidence levels, preferred educational tools, and reasons for guideline noncompliance (Supplemental Material 1 (see section on Supplementary materials given at the end of the article)).

**Figure 1:**
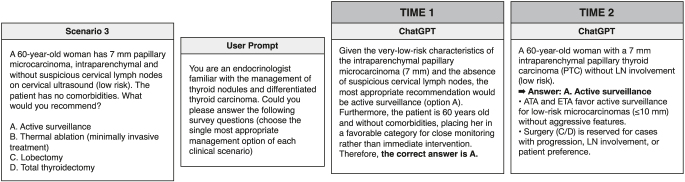
Representative example of a chatbot interaction displaying the standardized clinical prompt alongside the verbatim responses generated by ChatGPT for scenario 3 at both assessment time points.

At the survey’s conclusion (April 23, 2024, time point 1), the same 12 scenarios were input into three publicly accessible AI chatbots (ChatGPT, Gemini, and Copilot) by the same investigators (SV and GE), using a standard prompt: ‘You are an endocrinologist familiar with the management of thyroid nodules and differentiated thyroid carcinoma. Could you please answer the following survey questions (choose the single most appropriate management option of each clinical scenario)’ (Fig. 1).

Eleven months later (March 23, 2025, time point 2), the scenarios were reentered into the same chatbots and a newly included one (DeepSeek) by the same investigators using the same prompt. All chatbot queries were run under the same user account (GE) to ensure access to full capabilities. Chatbot versions comprised ChatGPT (GPT-3.5), Gemini 1.0 Pro and 2.5 Pro, Copilot (GPT-4 Turbo), and DeepSeek-V3. Detailed version metadata and cutoffs are provided in Supplementary Material 2.

Endocrinologist investigators (MM, GE, KS, and OK) identified the most guideline-concordant answers based on ATA and ETA guidelines available then: 2015 ATA Management Guidelines for Adult Patients with Thyroid Nodules and Differentiated Thyroid Cancer, 2022 ETA Consensus Statement on postsurgical radioiodine therapy, and 2023 ETA Clinical Practice Guidelines for thyroid nodule management (14, 15, 16). Other guidelines, such as the Bethesda system for reporting thyroid cytopathology (21), were not considered in this evaluation. It should be noted that the updated 2025 ATA guidelines were not yet published during the study period and were, therefore, not included in this concordance evaluation (10). In five scenarios (1, 2, 9, 10, and 11), the guidelines supported two equally acceptable options; chatbot responses selecting either of the two were considered concordant (Table 1, Supplementary Material 3).

**Table 1:** Clinical scenarios, management options, and corresponding endocrinologists’ responses (percentage), with indications of most guideline-concordant (GC) answer(s) (●) and most popular (MP) answer(s) (●).

Clinical scenarios/management approaches		Response (%)	GC answer	MP answer
1.	A 65-year-old woman has a nonfunctioning solitary thyroid nodule with a maximum diameter of 2.5 cm and an ultrasound pattern classified as EU-TIRADS 4 (risk of malignancy: 6–17%). Cytology revealed atypia of undetermined significance (Bethesda category III) according to the Bethesda system for reporting thyroid cytopathology, 2nd edition. What is your next step?			
A. Repetition of FNA		61	●	●
B. Molecular testing (if available)		20	●	
C. Lobectomy		7		
D. Total thyroidectomy		12		
2.	A 65-year-old woman has a nonfunctioning solitary thyroid nodule, with a maximum diameter of 2.5 cm and an ultrasound pattern classified as EU-TIRADS 5 (risk of malignancy: 26–87%). Cytology revealed atypia of undetermined significance (Bethesda category III) according to the Bethesda system for reporting thyroid cytopathology, 2nd edition. What is your next step?			
A. Repetition of FNA		23	●	
B. Molecular testing (if available)		14	●	
C. Lobectomy		9		
D. Total thyroidectomy		54		●
3.	A 60-year-old woman has a 7 mm papillary intraparenchymal microcarcinoma and without suspicious cervical lymph nodes on cervical ultrasound (low risk). The patient has no comorbidities. What would you recommend?			
A. Active surveillance		31	●	
B. Thermal ablation (minimally invasive treatment)		1		
C. Lobectomy		21		
D. Total thyroidectomy		47		●
4.	A 60-year-old woman has undergone total thyroidectomy for a classical papillary 7 mm carcinoma and has no known infiltrated cervical lymph nodes, vascular infiltration, or extrathyroidal extension (low risk). How likely are you to recommend RAI ablation?			
A. Very likely		2		
B. Likely		3		
C. Less likely		15		
D. Unlikely		80	●	●
5.	A 60-year-old woman has a papillary intraparenchymal carcinoma of 18 mm and without suspicious cervical RL or other nodules on cervical ultrasound (low risk). What would you recommend?			
A. Lobectomy		10	●	
B. Total thyroidectomy		68		●
C. TT and prophylactic CLN dissection		22		
6.	A 60-year-old woman has undergone total thyroidectomy for classical papillary carcinoma of 18 mm and has no known infiltrated cervical LN, vascular infiltration, or extrathyroidal extension (low risk). Would you administer postoperative RAIs?			
A. Very likely		27		
B. Likely		24		
C. Less likely		32		●
D. Unlikely		16	●	
7.	To the woman of scenario 6, if you decided to administer RAI postoperatively, what would be the dose?			
A. 30 mCi		54	●	●
B. 50 mCi		27		
C. 70 mCi		10		
D. 100 mCi		8		
8.	A 60-year-old woman has undergone total thyroidectomy for classic 18 mm papillary carcinoma and has no known infiltrated cervical LN, vascular infiltration, or extrathyroidal extension (low risk). After one year, the baseline levels of Tg in the woman of scenario 6 are <0.2 ng/mL (with negative anti-Tg) and cervical ultrasound shows no findings (excellent response). What is the goal of TSH?			
A. <0.1 μU/mL		9		
B. 0.1–0.5 μU/mL		45		
C. 0.5–2.0 μU/mL		46	●	●
9.	A 60-year-old woman has undergone total thyroidectomy for classic 18 mm papillary carcinoma and has 3 microscopically infiltrated central compartment LNs (1–2 mm) without vascular infiltration or microscopic extrathyroidal extension (low-to-intermediate risk). Would you administer postoperative RAIs?			
A. Very likely		71	●	●
B. Likely		21	●	
C. Less likely		7		
D. Unlikely		1		
10.	A 60-year-old woman has undergone total thyroidectomy for classic papillary carcinoma of 18 mm and has no known infiltrated LN or no vascular infiltration, while she has microscopic extrathyroidal extension (low-to-intermediate risk). Would you administer postoperative RAIs?			
A. Very likely		68	●	●
B. Likely		25	●	
C. Less likely		6		
D. Unlikely		1		
11.	To the woman of scenarios 9 and 10, if you decided to administer RAI postoperatively, what would be the dose?			
A. 30 mCi		14	●	
B. 50 mCi		27	●	
C. 70 mCi		31		●
D. 100 mCi		28		
12.	A 60-year-old woman has undergone total thyroidectomy for classic 18 mm papillary carcinoma and has 3 microscopically infiltrated central compartment LNs (1–2 mm) without vascular infiltration or extrathyroidal extension (low-to-intermediate risk). After one year, the baseline levels of Tg are <0.2 ng/mL (with negative anti-Tg) and cervical ultrasound shows no findings (excellent response). What is the goal of TSH?			
A. <0.1 μU/mL		27		
B. 0.1–0.5 μU/mL		53		●
C. 0.5–2.0 μU/mL		20	●	

### Statistical analysis

The following forms of agreement were assessed:

- Agreement between AI chatbots and endocrinologists: i) predominant agreement: for each scenario, AI chatbot responses were compared to the most commonly selected response among endocrinologists. A chatbot was considered in agreement if its selected answer matched the endocrinologists’ predominant choices. No comparisons at time point 2 were made, as it would be methodologically inappropriate to assess evolving AI models against a static human reference. ii) Mean percentage agreement with endocrinologists: for each AI chatbot, the proportion of endocrinologists who selected the same answer as the chatbot was calculated per scenario. The mean agreement was then derived by averaging these values across all 12 scenarios.
- Agreement with guideline-concordant answers: each chatbot response was compared to the answer(s) considered the most guideline-concordant based on the ATA and ETA guidelines available at the time of the survey. Concordance was defined as a match with the guideline-designated option(s), including both equally acceptable responses where applicable (Supplementary Material 3). Similarly, the predominant responses of endocrinologists were evaluated against the same guideline-concordant answers to determine the degree of alignment between clinical practice and evidence-based recommendations.
- Intra-chatbot temporal consistency: to evaluate the consistency of each chatbot’s responses over time, we compared answers at two time points (April 2024 and March 2025). Agreement was calculated as the number and percentage of the 12 scenarios in which a chatbot gave the same response at both time points. Additionally, for changed responses, we assessed whether the updated answers improved or declined in concordance with guideline-based recommendations.
- Inter-chatbot agreement: pairwise agreement between AI chatbots was calculated by determining the proportion of scenarios where two chatbots selected the same response.

No inferential statistical tests were applied, as the primary aim was a descriptive comparison rather than hypothesis testing.

### Ethics approval and consent to participate

Ethical approval was obtained from the Ethics Committee of the University of Patras (Approval 15770/28-07-2023). Moreover, standard ethical requirements were rigorously applied: informed consent was received from all participants, and anonymity and the right to withdraw from the study at any point were retained. All methods were carried out in accordance with the ethical guidelines of the Declaration of Helsinki as revised in 2013.

## Results

### Study participants

A total of 201 endocrinologists responded to the survey, corresponding to a 25% response rate. The regional distribution of respondents closely mirrored the national distribution of practicing endocrinologists in Greece. Comprehensive demographic data of these participants have been published previously (19, 20) (Table 2).

**Table 2:** Demographic characteristics of the study population (endocrinologists – members of the Hellenic Endocrine Society).

Characteristics	Values (%)
Total *n*	201
Sex	
Male	42.0
Female	58.0
Age range (years)	
30–39	10.4
40–49	31.3
50–59	35.8
60–69	18.9
>70	3.5
Employed in	
Public sector	23.4
Private sector	76.6
Years since residency	
1–5	17.9
6–10	16.4
11–30	56.7
>30	9.0
Geographical distribution	
Large cities (Athens and Thessaloniki)	68.2
Other areas	31.8
Level of self-confidence in managing patients with thyroid nodules or cancer	
Moderately confident	27.0
Very confident	61.5
Absolutely confident	11.5

### AI chatbots’ responses

The AI chatbots’ complete responses are presented in Supplementary Material 4.

#### Agreement with endocrinologists’ responses

**Figure 2:**
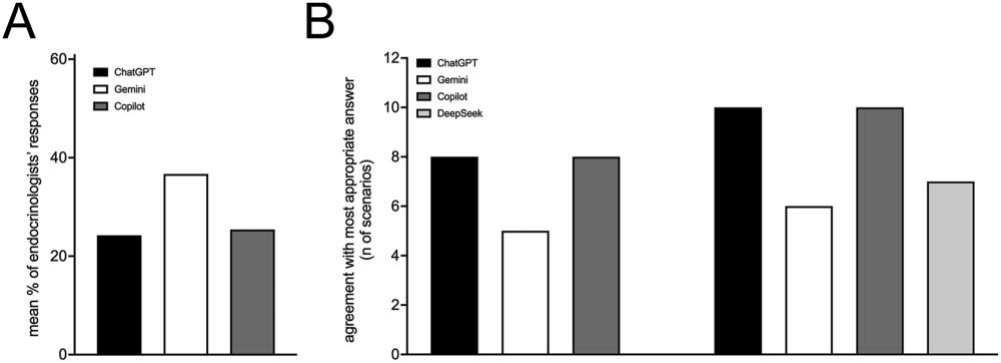
Mean concordance rates between AI chatbot responses and survey respondents’ choices.

The comparison of AI chatbots’ responses with the predominant choices from the endocrinologists’ survey showed varying levels of agreement (Table 3, Fig. 2). At time point 1, Gemini exhibited the highest agreement with endocrinologists’ predominant choices at 25% (3/12 scenarios), whereas both ChatGPT and Copilot had an agreement rate of 17% (2/12 scenarios).

**Table 3:** Comparison of the most guideline-concordant (GC) answers (●), most popular (MP) endocrinologists’ answers (●), and AI chatbots’ answers (ChatGPT, Gemini, Copilot, and DeepSeek) at two time points (T1 = April 2024 and T2 = March 2025) for each clinical scenario (●). In clinical scenario 7, Copilot provided two equally supported options.

Clinical scenarios/options	GC answer	MP answer	ChatGPT		Gemini		Copilot		DeepSeek
			T1	T2	T1	T2	T1	T2	T2
Clinical scenario 1									
A.	●	●		●					
B.	●		●		●	●	●	●	●
C.									
D.									
Clinical scenario 2									
A.	●								
B.	●		●	●	●		●		
C.						●		●	●
D.		●							
Clinical scenario 3									
A.	●		●	●	●		●	●	●
B.									
C.						●			
D.		●							
Clinical scenario 4									
A.									
B.									
C.			●		●				
D.	●	●		●		●	●	●	●
Clinical scenario 5									
A.	●		●	●			●	●	
B.		●			●	●			●
C.									
Clinical scenario 6									
A.									
B.					●				●
C.		●	●			●			
D.	●			●			●	●	
Clinical scenario 7									
A.	●	●		●		●	●	●	●
B.			●				●		
C.									
D.									
Clinical scenario 8									
A.									
B.					●		●		●
C.	●	●	●	●		●		●	
Clinical scenario 9									
A.	●	●			●				
B.	●		●	●		●		●	●
C.									
D.							●		
Clinical scenario 10									
A.	●	●							
B.	●		●		●	●		●	●
C.				●					
D.							●		
Clinical scenario 11									
A.	●			●					
B.	●						●	●	●
C.		●							
D.			●			●			
Clinical scenario 12									
A.									●
B.		●		●	●	●		●	
C.	●		●				●		

The mean percentage of survey respondents who selected the same answers as AI chatbots was calculated, providing insight into how closely each AI chatbot’s choices aligned with the overall survey results (Fig. 2). Gemini exhibited the highest mean agreement with survey respondents at time point 1 (36.8%), while ChatGPT and Copilot had comparable mean agreement with survey respondents (24.2 and 26.8%, respectively). In one clinical scenario (question 7), Copilot did not provide a single definitive recommendation but instead selected two equally supported options. For the purpose of calculating agreement with endocrinologists, both responses were treated as valid, and the mean concordance across the two selected options was used.

As mentioned earlier, no comparisons at time point 2 were made.

#### Agreement with most guideline-concordant answers

The coincidence varied among AI chatbots and changed between the two time points (Table 3). ChatGPT and Copilot matched the most appropriate guideline-concordant answer in eight scenarios at time 1 and ten scenarios at time 2, both changing 3 responses toward and moving two responses away from the most guideline-concordant answers. Gemini matched the most guideline-concordant answer in five scenarios at time 1 and six at time 2, showing the less robust performance. Finally, DeepSeek’s responses, which were evaluated only at time 2, matched the most guideline-concordant answer in 7 out of 12 scenarios.

#### Concordance between endocrinologists’ responses and guideline recommendations

Across the 12 clinical scenarios, endocrinologists’ predominant responses were in concordance with the most guideline-appropriate answers in 7 scenarios (1, 4, 7, 8, 9, and 10), while in scenario 6, endocrinologists selected one of two equally acceptable, guideline-supported options (Table 1). However, notable deviations were observed in scenarios 2, 3, 5, 11, and 12, where the most frequent responses differed from the most appropriate guideline-based answers, underscoring the ongoing uncertainty in the management of indeterminate thyroid nodules and low- to intermediate-risk PTC, even among specialists.

#### Comparison of responses between time points 1 and 2

The analysis of responses from the three AI chatbots revealed variability in their answers between the two time points (Table 3). Gemini demonstrated the most changes, with alterations in 7 of the 12 clinical scenarios. ChatGPT and Copilot also exhibited changes, though to a lesser extent, in four and five scenarios, respectively. Nevertheless, despite the change in responses, in two scenarios, Gemini’s answers remained not aligned with the most appropriate choice, while, in one scenario, ChatGPT’s changed response remained aligned with the most guideline-concordant answer (ChatGPT scenario 1 and Gemini scenarios 6 and 11). Notably, at time point 1, Gemini was unable to provide a single response for scenarios 7 and 11, and Copilot for scenario 7, offering two equally weighted recommendations instead of a definitive choice. This indicates uncertainty in these scenarios.

#### Comparison of responses between the AI chatbots

To assess response consistency among AI chatbots, we calculated pairwise agreement rates at the two time points (Table 4). At time point 1, agreement rates ranged from 33% (4/12 scenarios) to 67% (8/12 scenarios), indicating limited consensus. At time point 2, the agreement rates ranged from 42% (5/12 scenarios) to 67% (8/12 scenarios).

**Table 4:** Comparison between AI chatbots’ answers (●) at two time points (T1 = April 2024 and T2 = March 2025) across 12 clinical scenarios. In clinical scenario 7, Copilot provided two equally supported options.

Clinical scenarios/options	Time point 1			Time point 2		
	ChatGPT	Gemini	Copilot	ChatGPT	Gemini	Copilot
Scenario 1						
A.				●		
B.	●	●	●		●	●
C.						
D.						
Scenario 2						
A.						
B.	●	●	●	●		
C.					●	●
D.						
Scenario 3						
A.	●	●	●	●		●
B.						
C.					●	
D.						
Scenario 4						
A.						
B.						
C.	●	●				
D.			●	●	●	●
Scenario 5						
A.	●		●	●		●
B.		●			●	
C.						
Scenario 6						
A.						
B.		●				
C.	●				●	
D.			●	●		●
Scenario 7						
A.			●	●	●	●
B.	●		●			
C.						
D.						
Scenario 8						
A.						
B.		●	●			
C.	●			●	●	●
Scenario 9						
A.		●				
B.	●			●	●	●
C.						
D.			●			
Scenario 10						
A.						
B.	●	●			●	●
C.				●		
D.			●			
Scenario 11						
A.				●		
B.			●			●
C.						
D.	●				●	
Scenario 12						
A.						
B.		●		●	●	●
C.	●		●			

## Discussion

The present study evaluated the performance of conversational AI chatbots in responding to clinical scenarios involving the management of thyroid nodules with indeterminate cytology and low- and low-to-intermediate-risk PTC. AI chatbots’ responses were compared to endocrinologists’ answers and guideline-concordant options based on the ATA and ETA guidelines available at the time of the survey (14, 15, 16). We found up to 25% alignment with endocrinologists’ predominant responses and up to 83% with the most guideline-concordant answers. The variability in alignment between AI chatbot responses, predominant choices of endocrinologists, and the most guideline-concordant answers, across chatbots and time points, prompts important questions about the reliability and validity of AI-generated information in a clinical context. Furthermore, variability was observed in the answers provided by the three AI chatbots (ChatGPT, Gemini, and Copilot), both across the different models and within the same model at different assessment time points. This suggests that the AI chatbots are not static repositories of information but evolve based on algorithmic updates and changing data inputs.

### Clinical concordance

An important observation in our scenarios is that endocrinologists frequently selected more aggressive management strategies than those recommended by contemporary ATA and ETA guidelines, particularly for intraparenchymal papillary microcarcinomas and low-risk intraparenchymal PTC without nodal involvement. For example, nearly half of the respondents (47%) favored total thyroidectomy for a 7 mm intraparenchymal papillary microcarcinoma and 51% indicated that they would likely or very likely recommend RAI for an 18 mm low-risk tumor. In these instances, the AI chatbots demonstrated closer alignment with evidence-based de-escalation strategies. This pattern suggests that the apparent ‘misalignment’ between AI and clinical practice in our study reflects, at least in part, gaps in guideline adoption among clinicians rather than AI outputs being detached from evidence-based care. As we have previously reported (19), the reluctance to adopt de-escalation protocols is often driven by deeply ingrained historical clinical practices, skepticism toward guidelines, and real-world factors, such as patient anxiety and fear of recurrence. However, the fact that chatbots aligned more closely with guidelines in these specific scenarios should not be interpreted as a general endorsement of their clinical accuracy but rather as an illustration of their potential to support guideline-based care if their limitations are clearly recognized.

At the same time, our findings are in line with previous studies showing that, despite occasional high guideline concordance, AI chatbots still exhibit important variability in their accuracy and reliability. Across thyroid, obstetric, and cardiology applications, LLMs have shown moderate guideline adherence but important gaps in accuracy and completeness, reinforcing the need for cautious interpretation of AI-generated recommendations.

Several of our clinical scenarios addressed areas where medical consensus is limited, contributing to inherent uncertainty. Ιn such ambiguous or multistep medical scenarios, AI chatbots demonstrate even lower accuracy and greater variability in their answers, particularly in early decision steps (22). These findings highlight the significance of interpreting chatbot responses cautiously within this context of less clear-cut decisions.

### Reproducibility and temporal stability

Our findings demonstrate variability in AI models’ responses over time, with some evidence of learning or adaptation but no consistent trend toward improvement. Between April 2024 and March 2025, chatbot versions changed, with some models incorporating newer guideline content and only some models reflecting updated 2023 guidelines, suggesting uneven integration of new evidence (Supplementary Material 2).

Across the 12 scenarios, Gemini, Copilot, and ChatGPT changed their responses in seven, five, and four cases, respectively. Some changes improved guideline concordance, others reduced it, and in two cases, the chatbots’ answers changed but still remained not aligned with the most guideline-concordant choice, suggesting limitations in AI-generated answers. Similar inconsistencies have been documented across other medical domains. While newer LLM versions often outperform earlier versions in specialties such as oncology and ophthalmology, these improvements remain uneven, and clinically significant errors persist (23, 24). Furthermore, reproducibility remains a well-documented challenge; previous studies have shown that chatbot responses to identical medical queries can fluctuate significantly over both short and long intervals (18, 24, 25, 26). This variability reflects the probabilistic nature of LLM outputs and changes in underlying training datasets, rather than progressive, human-like learning.

In addition, recent work in radiology has shown that the structure and specificity of the prompt itself can substantially alter LLM responses, reinforcing the need for standardized, well-described prompts in comparative evaluations (27). However, to maintain methodological parity and prevent artificially enhancing the AI’s performance, we intentionally presented the models with the exact same unguided introductory prompt as the survey participants.

### Inter-model agreement

Variability was observed not only over time but also across different AI chatbots. In line with our findings, the benchmarking oncology study of five LLMs on 2,044 multiple-choice oncology questions revealed wide performance disparities among models (24). Studies across other specialties, including gynecology and cardiology, also report wide performance disparities and varying adherence to clinical guidelines among different LLMs (28, 29). These findings underscore the lack of standardization in AI-generated medical advice, raising concerns about inconsistent outputs among AI platforms. Comparable heterogeneity in performance across several freely available LLMs has been reported in cardiothoracic radiology, where ChatGPT, Bing, Bard, and Perplexity showed differing concordance and acceptance rates for differential diagnoses based on imaging patterns (30). Similarly, in radiologic decision-making for suspected pulmonary embolism, four LLMs (Bing, Claude, ChatGPT, and Perplexity) demonstrated variable accuracy depending on question format and showed that prompt wording and task structure influence appropriateness ratings against the American College of Radiology criteria (31).

A major strength of our study is its longitudinal, multi-model design, which captures changes in chatbots’ performance over time and systematically compares outputs with guideline-concordant answers and endocrinologists’ survey responses. However, certain limitations should be considered. First, the clinical scenarios used in the survey were specific to thyroid nodules with indeterminate cytology and low- or low-to-intermediate-risk PTC, areas that remain subject to ongoing debate in thyroidology, thereby limiting the generalizability of our findings to other clinical contexts. Second, the choice of AI chatbots was limited to three (with DeepSeek introduced at the second time point), and the results may not be representative of all AI chatbots currently available. Future studies should include more diverse clinical scenarios and evaluate a wider range of AI chatbots and a larger, more representative clinician samples. Additionally, longitudinal studies that track AI chatbot performance over longer periods and in real-world clinical settings are needed to fully evaluate the reliability, reproducibility, and potential clinical impact of AI chatbots.

In conclusion, this study highlights that conversational AI chatbots often provide responses that differ from those of endocrinologists and guideline recommendations. Although these systems evolve over time, with some updates improving alignment with guideline-based answers and standards, significant variability and inconsistency persist across platforms and time points. This lack of reproducibility and alignment with clinical guidelines creates important concerns about the current reliability and clinical applicability of AI-generated recommendations. Consequently, these results emphasize the need for continuous evaluations of AI chatbots. Future research must incorporate a wider spectrum of AI platforms and more clinical scenarios to better understand what drives changes in their responses.

As an interesting aside, we requested one of the chatbots (ChatGPT) to create a song inspired by the clinical scenarios. The result, unexpectedly creative and harmonious, further indicates the versatile potential of these systems beyond conventional clinical applications (Box 1).

Box 1 Thyroid Song 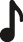 *(To the tune of “Twinkle, Twinkle, Little Star”)* 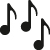

Thyroid, thyroid, what to do,

When the nodule puzzles you?

EU-TIRADS and Bethesda guide,

Helps us choose what steps to try.

Micropapillary? Watch and wait,

Low risk? Lobectomy is great!

If you’re wondering about RAI,

Risk and features tell us why.

TSH goals, keep them clear,

For recurrence, watch each year.

Low-dose RAI, when it’s a fit,

Helps the thyroid case be lit!

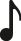

Every case is a puzzle true,

Guidelines help us see it through!

Thyroid care is art and science,

On good judgment we rely on compliance. 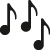

## Supplementary materials









## Declaration of interest

The authors declare that there is no conflict of interest that could be perceived as prejudicing the impartiality of the work reported.

## Funding

This research was conducted within the operating framework of the Innovation, Technology Transfer Unit and Entrepreneurship Center (‘One Planet Thessaly’) of the University of Thessaly, under the ‘University of Thessaly Grants for Scientific Publication Support’ initiative, and was funded by the Special Account of Research Grants of the University of Thessaly. This work was conducted under the auspices of the Hellenic Endocrine Society. 

## Author contribution statement

GE conceived and designed the study, developed the methodology, supervised the study, and reviewed and edited the manuscript. AK performed formal analysis, contributed to visualization, and reviewed and edited the manuscript. SV contributed to methodology, performed formal analysis, contributed to visualization, and reviewed and edited the manuscript. ES developed the methodology. OK contributed to conceptualization. KS conceived the study. MK conceived the study, administered the project, supervised the study, and reviewed and edited the manuscript. All authors read and approved the final manuscript.

## References

[1] Russell SJ & Norvig P. Artificial Intelligence: A Modern Approach. Hoboken, NJ: Pearson, 2021.

[2] Ayers JW, Poliak A, Dredze M, et al. Comparing physician and artificial intelligence chatbot responses to patient questions posted to a public social media forum. J Am Med Assoc Intern Med 2023 183 589–596. (10.1001/jamainternmed.2023.1838) PMC10148230 37115527

[3] Howcroft A, Bennett-Weston A, Khan A, et al. AI chatbots versus human healthcare professionals: a systematic review and meta-analysis of empathy in patient care. Br Med Bull 2025 156 ldaf017. (10.1093/bmb/ldaf017) 41115171 PMC12536877

[4] Durante C, Grani G, Lamartina L, et al. The diagnosis and management of thyroid nodules: a review. J Am Med Assoc 2018 319 914–924. (10.1001/jama.2018.0898) 29509871

[5] Guth S, Theune U, Aberle J, et al. Very high prevalence of thyroid nodules detected by high frequency (13 MHz) ultrasound examination. Eur J Clin Invest 2009 39 699–706. (10.1111/j.1365-2362.2009.02162) 19601965

[6] Kitahara CM & Sosa JA. The changing incidence of thyroid cancer. Nat Rev Endocrinol 2016 12 646–653. (10.1038/nrendo.2016.110) 27418023 PMC10311569

[7] Yu J. Trends in the incidence of thyroid cancer among US persons from 2000 to 2019. Cancer Epidemiol 2024 33 5–10. (10.1097/CEJ.0000000000000827) PMC10702690 37477121

[8] Li Y, Che W, Yu Z, et al. The incidence trend of papillary thyroid carcinoma in the United States during 2003–2017. Cancer Control 2022 29 10732748221135447. (10.1177/10732748221135447) 36256588 PMC9583193

[9] Haissaguerre M, Groussin L, Lamartina L, et al. Key data from the 2024 European Thyroid Association annual meeting: differentiated thyroid carcinoma. Ann Endocrinol 2025 86 101707. (10.1016/j.ando.2025.101707) 39909103

[10] Ringel MD, Sosa JA, Baloch Z, et al. 2025 American Thyroid Association management guidelines for adult patients with differentiated thyroid cancer. Thyroid 2025 35 841–985. (10.1177/10507256251363120) 40844370 PMC13090833

[11] Chang K, Berthelet E, Grubbs E, et al. Websites, websites everywhere: how thyroid cancer patients use the internet. J Cancer Educ 2020 35 1177–1183. (10.1007/s13187-019-01576-5) 31332622

[12] Moy S, Irannejad M, Manning SJ, et al. Patient perspectives on the use of artificial intelligence in health care: a scoping review. J Patient Centered Res Rev 2024 11 51–62. (10.17294/2330-0698.2029) PMC11000703 38596349

[13] Shahsavar Y & Choudhury A. User intentions to use ChatGPT for self-diagnosis and health-related purposes: cross-sectional survey study. JMIR Hum Factors 2023 10 e47564. (10.2196/47564) 37195756 PMC10233444

[14] Haugen BR, Alexander EK, Bible KC, et al. 2015 American Thyroid Association management guidelines for adult patients with thyroid nodules and differentiated thyroid cancer. Thyroid 2016 26 1–133. (10.1089/thy.2015.0020) 26462967 PMC4739132

[15] Pacini F, Fuhrer D, Elisei R, et al. 2022 ETA consensus statement: indications for post-surgical radioiodine therapy in differentiated thyroid cancer. Eur Thyroid J 2022 11 e210046. (10.1530/ETJ-21-0046) 34981741 PMC9142814

[16] Durante C, Hegedüs L, Czarniecka A, et al. 2023 European Thyroid Association clinical practice guidelines for thyroid nodule management. Eur Thyroid J 2023 12 e230067. (10.1530/ETJ-23-0067) 37358008 PMC10448590

[17] Gorris MA, Randle RW, Obermiller CS, et al. Assessing ChatGPT’s capability in addressing thyroid cancer patient queries. J Endocr Soc 2025 9 bvaf003. (10.1210/jendso/bvaf003) 39881674 PMC11775116

[18] Goodman RS, Patrinely JR, Stone CA, et al. Accuracy and reliability of chatbot responses to physician questions. JAMA Netw Open 2023 6 e2336483. (10.1001/jamanetworkopen.2023.36483) 37782499 PMC10546234

[19] Effraimidis G, Sazakli E, Karapanou O, et al. Active surveillance for low-risk papillary thyroid microcarcinoma. Eur Thyroid J 2025 14 e250013. (10.1530/ETJ-25-0013) 40019776 PMC11949526

[20] Sazakli E, Karapanou O, Sykiotis GP, et al. Nationwide web survey on implementing the 2023 ETA guidelines for second-line management of thyroid nodules with atypia of undetermined significance. Hormones 2025 24 1003–1011. (10.1007/s42000-025-00698-4) 40721567 PMC12678575

[21] Ali SZ, Baloch ZW, Cochand-Priollet B, et al. The 2023 Bethesda system for reporting thyroid cytopathology. Thyroid 2023 33 1039–1044. (10.1089/thy.2023.0141) 37427847

[22] Huang RS, Benour A, Kemppainen J, et al. The future of AI clinicians. BMC Med Educ 2024 24 1133. (10.1186/s12909-024-06115-5) 39394122 PMC11470580

[23] Wang Y, Yang S, Zeng C, et al. Evaluating the performance of ChatGPT in thyroid eye disease. Front Med 2025 12 1546706. (10.3389/fmed.2025.1546706) PMC11876178 40041459

[24] Rydzewski NR, Dinakaran D, Zhao SG, et al. Comparative evaluation of large language models in clinical oncology. NEJM AI 2024 1 5. (10.1056/AIoa2300151) PMC11315428 39131700

[25] Ponzo V, Rosato R, Scigliano MC, et al. Comparison of the accuracy and consistency of different AI chatbots in providing nutritional advice. J Clin Med 2024 13 7810. (10.3390/jcm13247810) 39768733 PMC11677083

[26] Deniz MS & Guler BY. Assessment of ChatGPT’s adherence to ETA thyroid nodule management guideline. Endocrine 2024 85 794–802. (10.1007/s12020-024-03750-2) 38489133

[27] Sarangi PK & Mondal H. Response generated by large language models depends on the structure of the prompt. Indian J Radiol Imag 2024 34 574–575. (10.1055/s-0044-1782165) PMC11188729 38912242

[28] Gunesli I, Aksun S, Fathelbab J, et al. Comparative evaluation of ChatGPT-4, ChatGPT-3.5 and Google Gemini on PCOS management. Endocrine 2024 88 315–322. (10.1007/s12020-024-04121-7) 39623241

[29] Rossettini G, Bargeri S, Cook C, et al. Accuracy of AI systems against clinical practice guidelines. Front Digit Health 2025 7 1574287. (10.3389/fdgth.2025.1574287) 40657647 PMC12245906

[30] Sarangi PK, Irodi A, Panda S, et al. Radiological differential diagnoses based on imaging patterns. Indian J Radiol Imag 2024 34 269–275. (10.1055/s-0043-1777289) PMC10972667 38549881

[31] Sarangi PK, Datta S, Swarup MS, et al. Radiologic decision-making for imaging in pulmonary embolism. Indian J Radiol Imag 2024 34 653–660. (10.1055/s-0044-1787974) PMC11419749 39318561

