# Supplementary material for: Promise and pitfalls of AI chatbots in complex decision-making for thyroid nodules and papillary thyroid cancer
Source: Eur Thyroid J. 2026 Apr 8;15(2):ETJ250385. doi: 10.1530/ETJ-25-0385 (PMC13087872; doi:10.1530/ETJ-25-0385)
Supplement: Supplementary file 1 [file supplementary_material_1.pdf]

A. Demographic data

1. Sex

a. Male

b. Female

c. Other

2. Age (years)

a. 30-39

b. 40-49

c. 50-59

d. 60-69

e. >70

3. Years since obtaining an endocrinology specialty

a. 1-5

b. 6-10

c. 11-30

d. >30

4. What is your workplace?

a. Private sector

b. Public sector

5. In which Prefecture do you practice your specialty? …………………………………………….

6. How confident do you feel about the management of patients with thyroid nodule or papillary thyroid carcinoma?

a. Not at all confident

b. A little bit confident

c. Moderately confident

d. Very confident

e. Absolutely confident

7. Which of the following do you think would help you manage patients with thyroid nodule or papillary thyroid carcinoma?

a. Conferences

b. Clinical tutorials - case studies in tertiary hospitals

c. Bibliography

B. Clinical scenarios

1. A 65-year-old woman has a non-functioning solitary thyroid nodule, with a maximum diameter of 2.5 cm and an ultrasound pattern classified as EU-TIRADS 4 (risk of malignancy: 6-17%). Cytology revealed Atypia of Undetermined Significance (Bethesda category III) according to The Bethesda System for Reporting Thyroid Cytopathology), 2nd edition. What is your next step?

A. repetition of FNA
B. molecular testing (if available)
C. lobectomy
D. total thyroidectomy.

2. A 65-year-old woman has a non-functioning solitary thyroid nodule, with a maximum diameter of 2.5 cm and an ultrasound pattern classified as EU-TIRADS 5 (risk of malignancy: 26-87%). Cytology revealed Atypia of Undetermined Significance (Bethesda category III) according to The Bethesda System for Reporting Thyroid Cytopathology), 2nd edition. What is your next step?

A. repetition of FNA
B. molecular testing (if available)
C. lobectomy
D. total thyroidectomy.

3. A 60-year-old woman has 7 mm papillary microcarcinoma, intraparenchymal and without suspicious cervical lymph nodes on cervical ultrasound (low risk). The patient has no comorbidities. What would you recommend?

A. Active surveillance
B. Thermal ablation (minimally invasive treatment)
C. Lobectomy
D. Total thyroidectomy

4. In a 60-year-old woman who has undergone total thyroidectomy for classical papillary 7mm carcinoma and has no known infiltrated cervical lymph nodes, vascular infiltration or extrathyroidal extension (low-risk). How likely are you to recommend Radioactive Iodine ablation?

A. Very likely
B. likely
C. Less likely
D. Unlikely

5. A 60-year-old woman has papillary carcinoma 18 mm, intraparenchymal and without suspicious cervical RL or other nodules on cervical ultrasound (low risk). What would you recommend?

A. Lobectomy
B. Total thyroidectomy
C. Total thyroidectomy and prophylactic central lymph node dissection

6. In a 60-year-old woman who has undergone total thyroidectomy for classical papillary carcinoma of 18 mm and has no known infiltrated cervical LN, vascular infiltration or extrathyroidal extension (low risk). Would you administer postoperative RAIs?

A. it is very likely
B. likely
C. less likely
D. unlikely

7. To the woman in question 6, if you decided to administer RAI postoperatively, what would be the dose?

A. 30 mCi
B. 50 mCi
C. 70 mCi
D. 100 mCi

8. A 60-year-old woman has undergone total thyroidectomy for classic 18mm papillary carcinoma and has no known infiltrated cervical LN, vascular infiltration or extrathyroidal extension (low risk). After one year the baseline levels of Tg are <0.2ng/mL (with negative anti-Tg) and cervical ultrasound without findings (excellent response). What is the goal of TSH:

A. TSH < 0.1μU/mL

B. TSH: 0.1 - 0.5μU/mL

C. TSH: 0.5-2.0 μU/mL

9. A 60-year-old woman has undergone total thyroidectomy for classic 18mm papillary carcinoma and has 3 microscopically infiltrated central compartment LNs (1-2mm) without vascular infiltration or microscopic extrathyroidal extension (low to intermediate risk). Would you administer postoperative RAIs?

A. it is very likely

B. likely

C. less likely

D. Unlikely

10. A 60-year-old woman has undergone total thyroidectomy for classic papillary carcinoma of 18 mm and has no known infiltrated LN or no vascular infiltration while she has microscopic extrathyroidal extension (low to intermediate risk). Would you administer postoperative RAIs?

A. it is very likely

B. likely

C. less likely

D. Unlikely

11. To the woman of question 9 and 10, if you decided to administer RAI postoperatively, what would be the dose?

A. 30mCi

B. 50mCi

C. 70mCi

D. 100mCi

12 . A 60-year-old woman has undergone total thyroidectomy for classic 18mm papillary carcinoma and has 3 microscopically infiltrated central compartment LNs (1-2mm) without vascular infiltration or extrathyroidal extension (low to intermediate risk). After one year the baseline levels of Tg are <0.2ng/mL (with negative anti-Tg) and cervical ultrasound without findings (excellent response). What is the goal of TSH:

A. TSH<0.1μU/mL
B. TSH:0.1-0,5μU/mL
C. TSH:0.5-2.0 μU/mL

C. Exploring Reasons for non-adherence

What is/are the main reason(s) for non-adherence to the guidelines regarding thyroid nodules or low-risk papillary thyroid carcinomas?

A. Insufficient information
B. I am not convinced by the guidelines, and I am concerned about my patient's safety
C. Inability to perform a reliable neck ultrasound
D. Inability to conduct molecular testing
E. lack of experienced surgeons across Greece
