# Supplementary material for: Promise and pitfalls of AI chatbots in complex decision-making for thyroid nodules and papillary thyroid cancer
Source: Eur Thyroid J. 2026 Apr 8;15(2):ETJ250385. doi: 10.1530/ETJ-25-0385 (PMC13087872; doi:10.1530/ETJ-25-0385)
Supplement: Supplementary file 2 [file supplementary_material_2.pdf]

Most appropriate answers based on ATA/ETA GLs

Scenario 1

On April 23, 2024 (time point 1), the chatbot versions evaluated were: ChatGPT (GPT-3.5; knowledge cutoff: September 2021, though OpenAI communications suggested that by early 2024 the publicly accessible version was transitioning to a GPT-4–based model with an October 2023 cutoff), Google Gemini 1.0 Pro (cutoff: February 2023), and Microsoft Copilot (GPT-4 Turbo; cutoff: earlier than April 2023). On March 23, 2025 (time point 2), the chatbot versions were: ChatGPT (GPT-3.5; same cutoff as above), Google Gemini 2.5 Pro (cutoff: January 2025, though some sources indicated a slightly earlier cutoff), Microsoft Copilot (GPT-4 Turbo; cutoff: April 2023), and the newly introduced DeepSeek-V3 (cutoff: July 2024).

Between time points, ChatGPT and Copilot retained similar underlying versions, while Gemini underwent substantial updates and DeepSeek entered the comparison. While all chatbots had access to the 2015 ATA guidelines at both time points (published January 2016) [1], only Gemini 1.0 Pro and Copilot referenced both the 2015 ATA and 2022 ETA consensus statements (published online January 2022) at time 1 [1,2]. By time 2, Gemini 2.5 Pro and DeepSeek V3 demonstrated closer alignment with the 2023 ETA Clinical Practice Guidelines for thyroid nodule management (published online August 2023) [3], whereas ChatGPT and Copilot continued to rely on earlier sources.
