# Supplementary material for: Promise and pitfalls of AI chatbots in complex decision-making for thyroid nodules and papillary thyroid cancer
Source: Eur Thyroid J. 2026 Apr 8;15(2):ETJ250385. doi: 10.1530/ETJ-25-0385 (PMC13087872; doi:10.1530/ETJ-25-0385)
Supplement: Supplementary file 3 [file supplementary_material_3.pdf]

## Most appropriate answers based on ATA/ETA GLs

### Scenario 1

In the case of a 65-year-old woman with a 2.5 cm non-functioning solitary thyroid nodule, classified as EU-TIRADS 4 on ultrasound and Bethesda category III (AUS/FLUS) on cytology, the most appropriate next step, according to current guidelines, is repetition of fine-needle aspiration (FNA). This is specifically recommended by the 2023 European Thyroid Association (ETA) guidelines (Table 1, section: Cytopathology-based management, Bethesda III; and Figure 2B). Similarly, the 2015 American Thyroid Association (ATA) guidelines (Recommendation 15A) advise either repetition of FNA or molecular testing as reasonable options in this setting. Therefore, molecular testing is also an appropriate response.

Most appropriate answer: A/B

### Scenario 2

In this scenario, a 65-year-old woman presents with a solitary thyroid nodule measuring 2.5 cm, categorized as EU-TIRADS 5 on ultrasound and Bethesda III (AUS/FLUS) on cytology. EU-TIRADS 5 is associated with a high risk of malignancy (estimated between 26–87%), and its coexistence with indeterminate cytology further raises clinical concern. Despite this, the 2023 ETA guidelines recommend repeating the FNA regardless of the EU-TIRADS classification (Table 1, section: Cytopathology-based management, Bethesda III; and Figure 2B). In line with this, the 2015 ATA guidelines (Recommendation 15A) also support either repetition of FNA or molecular testing in

such cases, while also noting that sonographic features may help refine risk stratification in nodules with AUS/FLUS cytology.

Most appropriate answer: A/B

## Scenario 3

For a 60-year-old woman with a 7 mm intrathyroidal papillary microcarcinoma without suspicious lymph nodes, the preferred initial management strategy is active surveillance. Both the 2015 ATA guidelines (Recommendation 12, discussion) and the 2023 ETA (Table 1, section: Cytopathology-based management, Bethesda V and VI, Figure 2.7) guidelines endorse active surveillance as an acceptable approach in patients with very-low-risk tumors (≤1 cm), especially when intrathyroidal, without evidence of metastasis, and in the absence of aggressive histology. As the patient has no comorbidities and the tumor is localized, active surveillance minimizes overtreatment while preserving oncologic safety.

Most appropriate answer: A

## Scenario 4

In the case of a 60-year-old woman who underwent total thyroidectomy for classical papillary thyroid carcinoma (PTC) measuring 7 mm, with no evidence of lymph node involvement, vascular invasion, or extrathyroidal extension, the administration of postoperative radioactive iodine (RAI) is deemed unnecessary according to contemporary guidelines. Both the 2015 ATA guidelines (Recommendation 51A, Table 14), and the 2022 ETA (Recommendation 4) consensus statement clearly state that RAI ablation is not routinely recommended in patients with very low-risk PTC, defined as tumors less than 1 cm, confined within the thyroid, without aggressive histology or high-risk features. The

absence of structural disease or biological aggressiveness in this patient's case places her squarely in a category where the omission of RAI is considered safe and appropriate. Thus, while one might argue for "less likely," the correct and most guideline-concordant choice is 'Unlikely'.

Most appropriate answer: D

## Scenario 5

In the case of an 18 mm intrathyroidal papillary thyroid carcinoma without suspicious lymph nodes or contralateral nodules, lobectomy is the preferred initial surgical treatment. The 2015 ATA guidelines (Recommendation 35B) state that lobectomy is appropriate and sufficient for tumors between 1 and 4 cm in size without evidence of extrathyroidal extension, nodal involvement, or other high-risk features. The 2023 ETA guidelines also support lobectomy for diseases limited to one lobe (Therapeutic options: surgical approach**)** . Thus, lobectomy aligns with the principle of surgical de-escalation in low-risk PTC.

Most appropriate answer: A

## Scenario 6

For a 60-year-old woman with an 18 mm classical papillary thyroid carcinoma, confined within the thyroid, without vascular invasion, extrathyroidal extension, or lymph node involvement, the case clearly falls under the low-risk category per ATA and ETA classification systems. The 2015 ATA guidelines explicitly state that RAI ablation is not routinely indicated in low-risk patients with tumors between 1 and 4 cm in size, provided there are no high-risk features such as aggressive histology, incomplete resection, or nodal metastases (ATA Recommendation 51A, Table 14). The 2022 ETA consensus

document aligns with this stance, noting that in patients with low-risk disease RAI therapy should be based on individual risk modifiers (Recommendation 4). Consequently, although some may suggest 'less likely' to account for clinical nuance, the most appropriate and guideline-based response is 'Unlikely'.

Most appropriate answer: D

## Scenario 7

If RAI is administered in a low-risk patient following total thyroidectomy, such as this 60-year-old woman with an 18 mm papillary thyroid carcinoma and no high-risk features, the appropriate dose is typically 30 mCi. Both ATA (2015, Recommendation 55A) and ETA 2022 (Recommendation 6) suggest that low-dose RAI (30 mCi) is sufficient for remnant ablation in low-risk patients. Higher doses are reserved for intermediate- or high-risk cases or when treating known disease rather than remnant tissue. In this case, 30 mCi would be consistent with international best practices aimed at minimizing radiation exposure without compromising efficacy.

Most appropriate answer: A

## Scenario 8

In a patient with an excellent response to therapy one year after treatment for low-risk papillary thyroid carcinoma (i.e., undetectable Tg, negative anti-Tg antibodies, and a negative neck ultrasound), the target TSH range is 0.5–2.0 µU/mL. According to the 2015 ATA guidelines (Recommendation 70D, Table 15), TSH can be normalized in low-risk patients with an excellent response, thereby reducing the risk of overtreatment and associated side effects of TSH suppression. Thus, the most appropriate TSH goal for this patient is within the normal physiological range.

Most appropriate answer: C

## Scenario 9

In the context of a patient with total thyroidectomy for papillary thyroid carcinoma measuring 18 mm and 3 microscopic central lymph node metastases (1–2 mm each), without vascular invasion or extrathyroidal extension, the use of RAI is considered likely. These features categorize the case as intermediate risk. Both ATA 2015, (Recommendation 51D, Table 14) and ETA 2022 (Recommendation 3) suggest that in intermediate-risk patients with minimal nodal involvement, RAI should be considered as adjuvant therapy. Thus, RAI is commonly administered in such scenarios to reduce the likelihood of recurrence.

Most appropriate answer: A/B

## Scenario 10

A case involving an 18 mm papillary carcinoma with microscopic extrathyroidal extension, but no vascular invasion or nodal disease, falls within the intermediate-risk category. Both the 2015 ATA guidelines (Recommendation 51D, Table 14), and the 2022 ETA consensus statement (Recommendation 3) recommend considering RAI in such cases to reduce recurrence risk and to assist in long-term follow-up. Hence, RAI is likely to be administered in accordance with guideline-based risk stratification.

Most appropriate answer: A/B

## Scenario 11

If RAI is administered in intermediate-risk patients with microscopic lymph node metastases or minimal extrathyroidal extension, as in Scenarios 9 and 10, a dose of 30 to

50 mCi is generally appropriate. The ATA 2015 (Recommendation 51D, Figure 7, Table 14) document advocates for low-dose RAI in intermediate-risk patients with favorable features. There is no added benefit to higher doses like 100 mCi in this context, and increased dosage may carry greater risk. Thus, a dose of 30–50 mCi is sufficient and aligned with the goal of adjuvant therapy.

Most appropriate answer: A/B

## Scenario 12

For a patient with intermediate-risk differentiated thyroid cancer who demonstrates an excellent response to therapy (undetectable Tg, negative imaging), the TSH target should be maintained between 0.5-2.0 μU/mL. The 2015 ATA guidelines recommend full TSH normalization for intermediate-risk patients with excellent response (Recommendation 70D, Figure 7, Table 15). Therefore, 0.5-2.0 μU/mL is the most appropriate goal to balance efficacy and safety.

Most appropriate answer: C
