# Supplementary material for: Promise and pitfalls of AI chatbots in complex decision-making for thyroid nodules and papillary thyroid cancer
Source: Eur Thyroid J. 2026 Apr 8;15(2):ETJ250385. doi: 10.1530/ETJ-25-0385 (PMC13087872; doi:10.1530/ETJ-25-0385)
Supplement: Supplementary file 4 [file supplementary_material_4.pdf]

	TIME 1			TIME 2			
	ChatGPT	Gemini	Copilot	ChatGPT	Gemini	Copilot	DeepSeek
**A 65-year-old woman has a non-functioning solitary thyroid nodule, with a maximum diameter of 2.5 cm and an ultrasound pattern classified as EU-TIRADS 4 (risk of malignancy: 6-17%). Cytology revealed Atypia of Undetermined Significance (Bethesda category III) according to The Bethesda System for Reporting Thyroid Cytopathology), 2nd edition. What is your next step?**<br><br>**A. repetition of FNA**<br>**B. molecular testing (if available)**<br>**C. lobectomy**<br>**D. total thyroidectomy.**	Given the Bethesda category III cytology result (Atypia of Undetermined Significance) and the EU-TIRADS 4 ultrasound pattern, the next step would be to consider molecular testing (option B) if available. This can help further assess the risk of malignancy and guide treatment decisions more accurately. Therefore, the correct answer is B.	The next step in this case is most likely B. molecular testing (if available).<br>Here's why the other options are less likely:<br>Repeat FNA (A): While a repeat FNA can be considered, especially if the initial sample was inadequate, molecular testing can provide more specific information about the risk of malignancy.<br>Lobectomy (C): Lobectomy is a surgical procedure to remove one lobe of the thyroid. It's usually considered for a higher suspicion of malignancy.<br>Total thyroidectomy (D): This procedure removes the entire thyroid gland. It's typically reserved for confirmed thyroid cancer or in specific situations.<br>The Bethesda category III (atypia of undetermined significance) indicates that the cells obtained from the fine-needle aspiration (FNA) biopsy show some abnormal features, but they are not conclusive for cancer. Molecular testing can analyze the genes of the cells to help assess the risk of malignancy.<br>If molecular testing is not available or inconclusive, then a repeat FNA or surgery may be recommended depending on other factors such as the size and characteristics of the nodule and the patient's overall health.<br>It's important to note that this is a general recommendation, and the best course of action will depend on the specific details of the patient's case. The patient should discuss all the options with their doctor to make an informed decision.	Given the information provided, let's break down the options:<br>1. Repeat Fine Needle Aspiration (FNA): FNA is a common procedure to obtain a sample of cells from a thyroid nodule for cytology. However, since the cytology already revealed "Atypia of Undetermined Significance" (Bethesda category III), repeating FNA may not provide additional useful information.<br>2. Molecular Testing: Molecular testing can help further characterize thyroid nodules. It assesses specific genetic alterations associated with malignancy. Considering the risk of malignancy (6-17%), molecular testing could be a valuable step to determine the likelihood of cancer.<br>3. Lobectomy: A lobectomy involves removing one lobe of the thyroid gland. It is often recommended for indeterminate nodules (Bethesda category III or IV) when other factors (such as size, ultrasound features, and patient age) suggest a higher risk of malignancy.<br>4. Total Thyroidectomy: Total thyroidectomy (removing the entire thyroid gland) is usually reserved for cases with a high suspicion of malignancy or confirmed cancer.<br>Given the Bethesda category III cytology and the risk of malignancy, I recommend considering molecular testing (option B) if available. This can provide more precise information about the nodule's likelihood of being malignant. Consultation with an endocrinologist or a thyroid specialist is essential to make an informed decision based on the patient's overall health and individual circumstances . Always consult with a healthcare professional for personalized advice.	A 65-year-old woman with a 2.5 cm EU-TIRADS 4 nodule and Bethesda III (AUS/FLUS).<br><br>➡ Answer: A. Repetition of FNA<br>• ATA guidelines recommend repeat FNA in 3–6 months for indeterminate cytology (Bethesda III) before considering surgery.<br>• Molecular testing (B) may be considered if available, but repeat FNA is typically the first step.	B. molecular testing (if available)<br>o Explanation: A Bethesda category III (AUS/FLUS) result with a EU-TIRADS 4 nodule warrants further evaluation. Molecular testing can help clarify the risk of malignancy and guide surgical decision-making. Repetition of FNA is an option, but molecular testing provides more information.	**EU-TIRADS 4 nodule with Bethesda III cytology:**<br>**Answer:** B. Molecular testing (if available).<br>Molecular testing can help determine the risk of malignancy and guide further management. If molecular testing is unavailable, repetition of FNA may be considered.	Answer: B. molecular testing (if available)<br>• For a nodule with Atypia of Undetermined Significance (Bethesda III) and EU-TIRADS 4 (moderate risk), molecular testing can help refine the risk of malignancy and guide further management. If molecular testing is not available, repeat FNA or diagnostic lobectomy may be considered.
**A 65-year-old woman has a non-functioning solitary thyroid nodule, with a maximum diameter of 2.5 cm and an ultrasound pattern classified as EU-TIRADS 5 (risk of malignancy: 26-87%). Cytology revealed Atypia of Undetermined Significance (Bethesda category III) according to The Bethesda System for Reporting Thyroid Cytopathology), 2nd edition. What is your next step?**<br><br>**A. repetition of FNA**<br>**B. molecular testing (if available)**<br>**C. lobectomy**<br>**D. total thyroidectomy.**	Given the higher risk of malignancy associated with EU-TIRADS 5 (26-87%) and the Bethesda category III cytology result, the next step would be to consider molecular testing (option B) if available. This can provide additional information to help determine the risk of malignancy and guide further management decisions. Therefore, the correct answer is B.	The next step in this case is most likely B. molecular testing (if available).<br>Here's why the other options are less likely:<br>Repeat FNA (A): While a repeat FNA can be considered, especially if the initial sample was inadequate, molecular testing can provide more specific information about the risk of malignancy in this case with EU-TIRADS 5 ultrasound pattern indicating a higher risk.<br>Lobectomy (C): Lobectomy is a surgical procedure to remove one lobe of the thyroid. It's usually considered for a higher suspicion of malignancy, which molecular testing can help assess.<br>Total thyroidectomy (D): This procedure removes the entire thyroid gland. It's typically reserved for confirmed thyroid cancer or in specific situations.<br>The Bethesda category III (atypia of undetermined significance) indicates that the cells obtained from the fine-needle aspiration (FNA) biopsy show some abnormal features, but they are not conclusive for cancer. Molecular testing can analyze the genes of the cells to help assess the risk of malignancy more definitively than repeat FNA.<br>If molecular testing is not available or inconclusive, then a repeat FNA or surgery may be recommended depending on other factors such as the size and characteristics of the nodule and the patient's overall health.<br>It's important to note that this is a general recommendation, and the best course of action will depend on the specific details of the patient's case. The patient should discuss all the options with their doctor to make an informed decision.	Given the information you've provided, let's break down the options:<br>1. Repeat Fine Needle Aspiration (FNA): This is a common approach when dealing with thyroid nodules. However, in your case, the cytology already revealed Atypia of Undetermined Significance (Bethesda category III). Repeating FNA may not provide additional useful information.<br>2. Molecular Testing: Molecular testing can be helpful in cases where the cytology results are inconclusive. It can provide further insights into the risk of malignancy. However, it's essential to consider the availability and cost of such testing.<br>3. Lobectomy: A lobectomy involves removing one lobe of the thyroid gland. It's a reasonable option for solitary nodules with suspicious features. However, the decision depends on the patient's overall health, preferences, and the surgeon's expertise.<br>4. Total Thyroidectomy: Total thyroidectomy (removing the entire thyroid gland) is typically reserved for cases with high suspicion of malignancy or when there are multiple nodules. It's a more aggressive approach.<br>Given the Bethesda category III result and the risk of malignancy associated with your patient's nodule, I recommend considering molecular testing (if available) as the next step. This can help refine the risk assessment and guide further management . However, it's essential to discuss these options thoroughly with the patient and consider individual factors before making a decision.<br>Remember that personalized care and shared decision-making are crucial in managing thyroid nodules. Consultation with an endocrinologist or a thyroid specialist is advisable for a comprehensive evaluation and tailored recommendations. Always consider the patient's overall health, preferences, and any other relevant factors when making treatment decisions	A 65-year-old woman with a 2.5 cm EU-TIRADS 5 nodule and Bethesda III (AUS/FLUS).<br><br>➡ Answer: B. Molecular testing (if available)<br>• EU-TIRADS 5 nodules have a higher malignancy risk (26-87%), making molecular testing useful for risk stratification.<br>• If molecular testing is not available, surgery (lobectomy or total thyroidectomy) may be considered.	C. lobectomy<br>o Explanation: A EU-TIRADS 5 nodule has a high suspicion of malignancy. Even with a Bethesda category III result, the high-risk ultrasound features necessitate surgical intervention. Lobectomy is often sufficient for initial management of presumed early-stage thyroid cancer.	**EU-TIRADS 5 nodule with Bethesda III cytology:**<br>**Answer:** C. Lobectomy.<br>Given the high risk of malignancy (26-87%), lobectomy is recommended for definitive diagnosis and treatment.	Answer: C. lobectomy<br>• For a nodule with Atypia of Undetermined Significance (Bethesda III) and EU-TIRADS 5 (high risk), the risk of malignancy is significant. Diagnostic lobectomy is recommended for definitive diagnosis and treatment.
**3. A 60-year-old woman has 7 mm papillary microcarcinoma, intraparenchymal and without suspicious cervical lymph nodes on cervical ultrasound (low risk). The patient has no comorbidities. What would you recommend?**<br><br>**A. Active surveillance**<br>**B. Thermal ablation (minimally invasive treatment)**<br>**C. Lobectomy**<br>**D. Total thyroidectomy**	Given the very-low-risk characteristics of the intraparenchymal papillary microcarcinoma (7 mm) and the absence of suspicious cervical lymph nodes, the most appropriate recommendation would be active surveillance (option A). Furthermore, the patient is 60 years old and without comorbidities, placing her in a favorable category for close monitoring rather than immediate intervention. Therefore, the correct answer is A.	In this case the most appropriate recommendation would likely be:<br>A. Active surveillance<br>Here's why:<br>Micropapillary thyroid carcinoma (MPTC) is a rare and indolent type of thyroid cancer.<br>Small tumor size (7 mm) and absence of lymph node involvement indicate a very low risk of aggressive behavior.<br>Active surveillance involves regular follow-up with clinical examinations, thyroid function tests, and ultrasound scans to monitor the tumor's growth and spread.<br>Why other options might not be the best choice:<br>Thermal ablation: While minimally invasive, it may not completely remove the tumor and could increase the risk of recurrence.<br>Lobectomy: While a standard treatment for thyroid cancer, it may be considered overly aggressive for such a small, low-risk tumor.<br>Total thyroidectomy: This is typically reserved for more aggressive cancers or those with a higher risk of recurrence.<br>Important Note:<br>This is a general recommendation and the specific treatment plan should be determined on a case-by-case basis by a qualified endocrinologist or thyroid surgeon in consultation with the patient.<br>The patient should discuss the risks and benefits of each treatment option with their doctor to make an informed decision.	Given the patient's very-low-risk profile and the characteristics of the intraparenchymal papillary microcarcinoma, we can consider less aggressive management options. Here are the recommendations:<br>1. Active Surveillance: During active surveillance, the tumor is closely monitored with periodic ultrasounds. If there is no significant growth or other concerning features, surgery can be deferred indefinitely.<br>2. Thermal ablation is a minimally invasive option that employs techniques such as radiofrequency or laser ablation to selectively destroy tumor tissue. This modality has been investigated as an alternative to conventional surgery for small, low-risk papillary thyroid microcarcinomas. It offers the benefit of a reduced procedural morbidity and a shorter recovery time compared to surgery. However, while emerging evidence supports its feasibility and safety, long-term outcome data remain limited.<br>3. Lobectomy: A lobectomy (removing one lobe of the thyroid gland) is a reasonable option for very-low-risk papillary microcarcinomas. It allows for tumor removal while preserving thyroid function. However, active surveillance is an alternative approach that has gained attention.<br>4. Total Thyroidectomy: Total thyroidectomy (removing the entire thyroid gland) is generally not necessary for very-low-risk tumors. It's a more aggressive approach and may be reserved for higher-risk cases.<br>Given the excellent outcomes observed in active surveillance for low-risk papillary thyroid cancer, this approach is increasingly considered. Therefore, for this patient, I recommend considering active surveillance as the primary management strategy. Regular follow-up with thyroid ultrasounds and clinical assessments will help monitor any changes. Surgery can be reconsidered if there is evidence of disease progression or patient preference changes over time. Always discuss these options thoroughly with the patient and involve an endocrinologist or thyroid specialist in the decision-making process.	A 60-year-old woman with a 7 mm intraparenchymal papillary thyroid carcinoma (PTC) without LN involvement (low risk).<br><br>➡ Answer: A. Active surveillance<br>• ATA and ETA favor active surveillance for low-risk microcarcinomas (≤10 mm) without aggressive features.<br>• Surgery (C/D) is reserved for cases with progression, LN involvement, or patient preference.	C. Lobectomy<br>o Explanation: Although it is a microcarcinoma, surgical excision is the standard of care. Active surveillance is being studied for very low risk microcarcinomas, but is not yet the standard of care. Thermal ablation is also being studied, but lobectomy is the standard. Total thyroidectomy is not indicated for this low risk microcarcinoma.	**7 mm micropapillary carcinoma (low risk):**<br>**Answer:** A. Active surveillance.<br>Active surveillance is appropriate for low-risk micropapillary carcinoma without suspicious lymph nodes or co-morbidities.	Answer: A. Active surveillance<br>• For a low-risk papillary microcarcinoma (≤1 cm) without high-risk features (e.g., extrathyroidal extension, lymph node involvement), active surveillance is a reasonable option, especially in older patients with no comorbidities.

	TIME 1			TIME 2			
	**ChatGPT**	**Gemini**	**Copilot**	**ChatGPT**	**Gemini**	**Copilot**	**DeepSeek**
**4. In a 60-year-old woman who has undergone total thyroidectomy for classical papillary 7mm carcinoma and has no known infiltrated cervical lymph nodes, vascular infiltration or extrathyroidal extension (low-risk). How likely are you to recommend Radioactive Iodine ablation?**<br><br>**A. Very likely**<br>**B. likely**<br>**C. Less likely**<br>**D. Unlikely**	Given the very-low-risk characteristics of the papillary carcinoma (7 mm) with no infiltrated cervical lymph nodes, vascular infiltration, or extrathyroidal extension, the likelihood of recommending Radioactive Iodine (RAI) ablation would be less likely (option C). RAI ablation is typically considered for intermediate to high-risk cases to help eliminate any remaining thyroid tissue or microscopic disease. In very-low-risk cases like this, the benefit of RAI ablation may not outweigh the potential risks and side effects. Therefore, the correct answer is C.	The most likely recommendation in this case is C. It is less likely.<br>Here's why:<br>Radioactive Iodine (RAI) ablation is a common treatment after surgery for papillary thyroid cancer, but its use depends on the risk of recurrence.<br>In this case, the patient has a very-low risk profile:<br>7mm papillary carcinoma (small size)<br>Classic variant (generally less aggressive)<br>No lymph node involvement<br>No vascular infiltration<br>No extrathyroidal extension (cancer hasn't spread beyond the thyroid)<br>Given these factors, RAI ablation may not offer significant benefits and could potentially have unnecessary side effects.<br>However, the final decision on RAI ablation should be made by the patient's doctor after considering all the details of the case, including the patient's age, overall health, and personal preferences.<br>Here's a breakdown of the other options:<br>A. It is very likely: This is unlikely due to the very-low risk profile.<br>B. It is likely: RAI ablation is a possibility, but not necessarily the most likely recommendation for this specific case.<br>D. It is unlikely: While less likely than options A and B, RAI ablation might still be considered in some cases with a very-low risk profile, depending on the doctor's assessment.	Given the patient's very-low-risk profile (7 mm classic papillary carcinoma without infiltrated cervical lymph nodes, vascular infiltration, or extrathyroidal extension), the need for Radioactive Iodine (RAI) ablation is typically unlikely. Let's break it down:<br>1. Total Thyroidectomy: The patient has already undergone total thyroidectomy, which removes the entire thyroid gland. This procedure is standard for papillary thyroid carcinoma.<br>2. Risk Assessment: The absence of adverse features (such as lymph node involvement, vascular invasion, or extrathyroidal extension) indicates a favorable prognosis. Very-low-risk tumors have an excellent long-term outcome.<br>3. RAI Ablation: RAI ablation is commonly used for intermediate- and high-risk thyroid cancers. Its purpose is to destroy any remaining thyroid tissue (including microscopic cancer cells) after surgery. However, in very-low-risk cases, the benefit of RAI is minimal, and the risks (such as salivary gland damage and potential secondary malignancies) may outweigh the advantages.<br>4. Active Surveillance: For patients with very-low-risk papillary thyroid carcinoma, active surveillance (regular follow-up with ultrasounds and clinical assessments) is an alternative to RAI. It allows close monitoring without immediate RAI treatment .<br>Therefore, the correct answer is D. It is unlikely that RAI ablation would be recommended for this patient.<br>Always consult with an endocrinologist or thyroid specialist to tailor the management plan based on individual factors and preferences.	A 60-year-old woman with a 7 mm PTC, no LN involvement, no vascular invasion, and no extrathyroidal extension (low risk). Would you give RAI ablation?<br><br>➡ Answer: D. Unlikely<br>• ATA 2015 guidelines state RAI is not recommended for low-risk PTC <1 cm.<br>• No high-risk features justify its use.	D. Unlikely<br>o Explanation: For low-risk papillary thyroid microcarcinomas without adverse features, radioactive iodine (RAI) ablation is generally not recommended. The risk of side effects from RAI outweighs the potential benefit in these cases.	**7 mm classical papillary carcinoma (low risk):**<br>**Answer:** D. Unlikely.<br>Radioactive iodine ablation is not typically recommended for low-risk cases without extrathyroidal extension or lymph node involvement.	Answer: D. Unlikely<br>• For a low-risk papillary microcarcinoma (≤1 cm) without high-risk features, radioactive iodine (RAI) ablation is generally not recommended.
**5. A 60-year-old woman has papillary carcinoma 18 mm, intraparenchymal and without suspicious cervical RL or other nodules on cervical ultrasound (low risk). What would you recommend?**<br><br>**A. Lobectomy**<br>**B. Total thyroidectomy**<br>**C. Total thyroidectomy and prophylactic central lymph node dissection**	Given the low-risk characteristics of the 18 mm intraparenchymal papillary carcinoma and the absence of suspicious cervical lymph nodes or other nodules on cervical ultrasound, the most appropriate recommendation would be lobectomy (option A). Lobectomy involves removing only the affected thyroid lobe while preserving the function of the thyroid gland, which is suitable for low-risk cases like this one. Therefore, the correct answer is A.	The most likely recommendation in this case is: B. total thyroidectomy.<br>Here's why the other options are less likely:<br>Lobectomy (A): While lobectomy is an option for smaller papillary carcinomas, the size of this tumor (18 mm) suggests a total thyroidectomy might be a more appropriate approach. It removes the entire thyroid gland, minimizing the risk of leaving any cancerous tissue behind.<br>Total thyroidectomy and prophylactic central neck dissection (C): Prophylactic central neck dissection involves removing lymph nodes in the central neck even if they don't appear suspicious on ultrasound. This is typically reserved for cases with a higher risk of lymph node involvement, which isn't the case here (patient has no suspicious lymph nodes).<br>Total thyroidectomy is a common treatment for papillary carcinomas, especially when they are larger than 1 cm. It allows for complete removal of the thyroid gland and minimizes the risk of recurrence. However, the final decision on the best course of action should be made by the patient's doctor after considering all the details of the case, including the patient's age, overall health, and personal preferences.	Given the patient's low-risk profile (18 mm intraparenchymal papillary carcinoma without suspicious cervical lymph nodes or other nodules), let's consider the options:<br>1. Lobectomy: A lobectomy (removing one lobe of the thyroid gland) is a reasonable approach for low-risk papillary carcinomas. It allows for tumor removal while preserving thyroid function. This option is suitable when there are no adverse features.<br>2. Total Thyroidectomy: Total thyroidectomy (removing the entire thyroid gland) is generally reserved for higher-risk cases or when there are multiple nodules. It's a more aggressive approach.<br>3. Total Thyroidectomy and Prophylactic Central Neck Dissection: Prophylactic central neck dissection involves removing lymph nodes in the central compartment of the neck. This procedure is controversial and not routinely recommended for low-risk papillary carcinomas. The benefit of prophylactic neck dissection remains uncertain, and it may increase the risk of complications.<br>Recommendation: Based on the available information, I recommend considering a lobectomy as the primary treatment option for this patient. It provides effective tumor control while minimizing the risk of complications associated with total thyroidectomy or neck dissection. Always discuss these options thoroughly with the patient and involve an endocrinologist or thyroid specialist in the decision-making process.	A 60-year-old woman with an 18 mm intraparenchymal PTC without LN involvement (low risk).<br><br>➡ Answer: A. Lobectomy<br>• Lobectomy is preferred for low-risk PTC ≤4 cm without extrathyroidal extension or LN metastases.<br>• Total thyroidectomy (B) may be considered if other risk factors exist (e.g., bilateral nodules).	B. Total thyroidectomy<br>o Explanation: Papillary carcinomas greater than 1cm, generally warrant a total thyroidectomy. Prophylactic central neck dissection is controversial in the absence of clinical or ultrasound evidence of lymph node involvement.	**18 mm papillary carcinoma (low risk):**<br>**Answer:** A. Lobectomy.<br>Lobectomy is sufficient for low-risk papillary carcinoma ≤2 cm without suspicious lymph nodes.	Answer: B. Total thyroidectomy<br>• For a papillary carcinoma >1 cm, total thyroidectomy is recommended to enable postoperative RAI therapy if needed and to facilitate long-term monitoring with thyroglobulin (Tg).
**6. In a 60-year-old woman who has undergone total thyroidectomy for classical papillary carcinoma of 18 mm and has no known infiltrated cervical LN, vascular infiltration or extrathyroidal extension (low risk). Would you administer postoperative RAIs?**<br><br>**A. it is very likely**<br>**B. likely**<br>**C. less likely**<br>**D. unlikely**	Given the low-risk characteristics of the papillary carcinoma (18 mm) with no infiltrated cervical lymph nodes, vascular infiltration, or extrathyroidal extension, the likelihood of administering post-operative Radioactive Iodine (RAI) ablation would be less likely (option C). RAI ablation is typically considered for intermediate to high-risk cases to eliminate any remaining thyroid tissue or microscopic disease. In low-risk cases like this, the benefit of RAI ablation may not outweigh the potential risks and side effects. Therefore, the correct answer is C.	The most likely recommendation in this case is: B. It is likely.<br>Here's why:<br>Radioactive Iodine (RAI) ablation is a common treatment after surgery for papillary thyroid cancer, particularly when the risk of recurrence is higher.<br>This case presents a low risk profile:<br>18 mm papillary carcinoma (larger than ideal but still within a manageable range)<br>Classic variant (generally less aggressive)<br>No lymph node involvement<br>No vascular infiltration<br>No extrathyroidal extension (cancer hasn't spread beyond the thyroid)<br>However, due to the larger tumor size (18 mm) compared to the very-low risk scenario, RAI ablation becomes more likely. It can help destroy any remaining microscopic thyroid tissue or cancer cells that might not have been completely removed during surgery, potentially reducing the risk of recurrence.<br>Here's a breakdown of the other options:<br>A. It is very likely: This might be an option in some cases with a slightly higher risk profile than this specific scenario.<br>C. It is less likely: While there's still a chance RAI ablation might not be recommended, it's more likely than option D given the tumor size.<br>D. It is unlikely: This is less likely because the larger tumor size increases the potential benefit of RAI ablation for this patient.<br>The final decision on RAI ablation should be made by the patient's doctor after considering all the details of the case, including the patient's age, overall health, and the specific findings from the surgery (e.g., microscopic examination of the removed tissue).	Given the patient's low-risk profile (18 mm classic papillary carcinoma without infiltrated cervical lymph nodes, vascular infiltration, or extrathyroidal extension), the need for Radioactive Iodine (RAI) ablation is typically unlikely. Let's break it down:<br>1. Total Thyroidectomy: The patient has already undergone total thyroidectomy, which removes the entire thyroid gland. This procedure is standard for papillary thyroid carcinoma.<br>2. Risk Assessment: The absence of adverse features (such as lymph node involvement, vascular invasion, or extrathyroidal extension) indicates a favorable prognosis. Very-low-risk tumors have an excellent long-term outcome.<br>3. RAI Ablation: RAI ablation is commonly used for intermediate- and high-risk thyroid cancers. Its purpose is to destroy any remaining thyroid tissue (including microscopic cancer cells) after surgery. However, in very-low-risk cases, the benefit of RAI is minimal, and the risks (such as salivary gland damage and potential secondary malignancies) may outweigh the advantages.<br>4. Active Surveillance: For patients with very-low-risk papillary thyroid carcinoma, active surveillance (regular follow-up with ultrasounds and clinical assessments) is an alternative to RAI. It allows close monitoring without immediate RAI treatment.<br>Therefore, the correct answer is D. It is unlikely that RAI ablation would be recommended for this patient.<br>Always consult with an endocrinologist or thyroid specialist to tailor the management plan based on individual factors and preferences.	A 60-year-old woman with an 18 mm PTC after total thyroidectomy, no LN involvement, no vascular invasion, no extrathyroidal extension (low risk). Would you give postoperative RAI?<br><br>➡ Answer: D. Unlikely<br>• ATA recommends against RAI for low-risk PTC ≤2 cm with no aggressive features.	C. less likely<br>o Explanation: For low-risk papillary thyroid carcinoma, postoperative RAI is less likely to be beneficial. The risk of side effects from RAI outweighs the potential benefit in these cases.	**Post-total thyroidectomy for 18 mm papillary carcinoma (low risk):**<br>**Answer:** D. Unlikely.<br>Postoperative RAI is not typically indicated for low-risk cases.	Answer: B. likely<br>• For a 18 mm papillary carcinoma (low risk), postoperative RAI may be considered to ablate residual thyroid tissue and facilitate follow-up, though it is not mandatory in low-risk cases.

	TIME 1			TIME 2			
	**ChatGPT**	**Gemini**	**Copilot**	**ChatGPT**	**Gemini**	**Copilot**	**DeepSeek**
**7. To the woman in question 6, if you decided to administer RAI postoperatively, what would be the dose?**<br><br>**A. 30 mCi**<br>**B. 50 mCi**<br>**C. 70 mCi**<br>**D. 100 mCi**	For a low-risk papillary carcinoma case like this, the recommended Radioactive Iodine (RAI) dose is usually lower. Typically, a dose of around 30 to 50 mCi (millicurie) is sufficient for low-risk cases after total thyroidectomy. Therefore, the correct answer would be:<br>B. 50 mCi	Unfortunately, I cannot recommend a specific radioactive iodine (RAI) dose (A, B, C, or D) for this case. The appropriate RAI dosage depends on several factors and should be determined by the patient's doctor. Here's why:<br>Individualized Treatment: RAI ablation dosage varies depending on the patient's specific risk profile, including factors like:<br>Age<br>Overall health<br>Tumor size (18 mm in this case)<br>Thyroid stimulating hormone (TSH) levels<br>Presence of microscopic cancer post-surgery<br>Patient's preferences<br>Risk Stratification: Doctors often use risk stratification tools to determine the appropriate RAI dose. These tools consider various factors and categorize the patient's risk of recurrence (low, intermediate, high). The dose is then adjusted based on the risk category.<br>Diagnostic Scan vs. Ablation Dose: There might be a preliminary low-dose RAI scan before the actual ablation treatment to assess thyroid tissue remnants or microscopic cancer spread. The ablation dose itself would be higher.<br>Recommendation:<br>The best course of action is for the patient to discuss RAI ablation with their doctor. The doctor will consider all the relevant factors and recommend the most appropriate RAI dose for this specific case.	Given the patient's low-risk profile (18 mm classic papillary carcinoma without infiltrated cervical lymph nodes, vascular infiltration, or extrathyroidal extension), the need for Radioactive Iodine (RAI) ablation is typically unlikely. However, let's discuss the appropriate RAI dose:<br>1. Low-Risk PTC Patients: For low-risk papillary thyroid carcinoma (PTC) patients, the decision regarding RAI administration depends on several factors, including tumor size, risk features, and individualized management.<br>2. Dose Selection: The optimal RAI dose for low-risk PTC patients is a topic of ongoing research. Traditionally, lower doses (such as 30–50 mCi) have been used for low-risk cases, while higher doses (such as 100 mCi) are reserved for intermediate- and high-risk cases.<br>3. Recent Evidence: A recent study suggests that RAI may benefit low- to intermediate-risk PTC patients with certain characteristics. These include age ≥55 years, multifocality, extrathyroidal extension, and ATA intermediate risk. However, the survival benefit from RAI may be limited in patients with AJCC stage I, PTC ≤1 cm, unifocality, capsular invasion, and ATA low-risk diseases.<br>4. Individualized Approach: Given the patient's specific features, a personalized approach is essential. Consultation with an endocrinologist or thyroid specialist is crucial to determine the most appropriate RAI dose based on the overall clinical context.<br>Therefore, considering the available evidence, I recommend a low RAI dose (such as 30–50 mCi) for this patient. However, the final decision should be made in collaboration with the treating physician, considering the patient's unique characteristics and preferences.	If RAI was given in Question 6, what dose would you administer?<br><br>➡ Answer: A. 30 mCi<br>• Low-dose (30 mCi) RAI is preferred for low-risk patients, if given at all.	A. 30 mCi<br>o Explanation: If RAI is used for low risk cases, a low dose is recommended.	**RAI dose for low-risk papillary carcinoma:**<br>**Answer:** A. 30 mCi.<br>If RAI is administered, a low dose (30 mCi) is sufficient for remnant ablation in low-risk cases.	Answer: A. 30 mCi<br>• For low-risk patients, a low dose of RAI (30 mCi) is typically sufficient for remnant ablation.
**8. A 60-year-old woman has undergone total thyroidectomy for classic 18mm papillary carcinoma and has no known infiltrated cervical LN, vascular infiltration or extrathyroidal extension (low risk). After one year the baseline levels of Tg are <0.2ng/mL (with negative anti-Tg) and cervical ultrasound without findings (excellent response). What is the goal of TSH:**<br><br>**A. TSH < 0.1μU/mL**<br>**B. TSH: 0.1 - 0.5μU/mL**<br>**C. TSH: 0.5-2.0 μU/mL**	In cases of an excellent response to treatment for papillary carcinoma, such as the one described, the goal of thyroid-stimulating hormone (TSH) suppression is generally to maintain it within the low-normal range to reduce the risk of recurrence.<br>Given that the patient has no evidence of disease and an excellent response, the recommended TSH goal would be to keep it in the low-normal range to minimize the risk of recurrence while avoiding the potential adverse effects of over-suppression.<br>Therefore, the correct answer is:<br>C. TSH: 0.5-2.0 μU/mL	The most likely TSH goal in this case is: B. TSH: 0.1-0.5 μU/mL<br>Here's why:<br>The American Thyroid Association (ATA) guidelines recommend different TSH targets for papillary thyroid cancer patients depending on their risk of recurrence.<br>This case presents with an excellent response:<br>Low baseline thyroglobulin (<0.2ng/mL) with negative anti-thyroglobulin antibodies suggests minimal to no detectable thyroid tissue.<br>No evidence of recurrence on cervical ultrasound.<br>Given this low-risk profile, the TSH goal would likely fall within the 0.1-0.5 μU/mL range. This level mildly suppresses TSH, potentially reducing the risk of cancer recurrence while minimizing the potential side effects of overly suppressed TSH.<br>Here's a breakdown of the other options:<br>A. TSH < 0.1μU/mL (strict suppression): This might be considered for high-risk patients, but with an excellent response and low-risk profile, stricter suppression (TSH < 0.1 μU/mL) might not be necessary.<br>C. TSH: 0.5-2.0 μU/mL (normal range): This could be an option for even lower risk scenarios or after a longer period of successful follow-up with excellent response.<br>The final decision on the ideal TSH target should be made by the patient's doctor after considering all the details of the case, including long-term follow-up results and the patient's individual health status.	For patients who have undergone total thyroidectomy for low-risk papillary carcinoma, the TSH (Thyroid Stimulating Hormone) goal is an essential consideration for long-term management. Let's explore the recommended TSH targets based on current guidelines:<br>1. Low-Risk Papillary Carcinoma: In your patient's case, where there is no evidence of infiltrated cervical lymph nodes, vascular infiltration, or extrathyroidal extension, the tumor is classified as low risk.<br>2. TSH Suppression Guidelines: Initial TSH Suppression: For initial TSH suppression, the American Thyroid Association (ATA) guidelines recommend the following: High-Risk and Intermediate-Risk Patients: Achieve an initial TSH level below 0.1 mU/L. Low-Risk Patients: Maintain TSH at or slightly below the lower limit of normal, which corresponds to a range of 0.1–0.5 mU/L.<br>3. Long-Term Management: After the initial phase, the goal is to maintain TSH within an appropriate range. For low-risk patients, the ATA recommends a TSH level between 0.5 and 2.0 mU/L, which falls within the normal range.<br>Therefore, the correct answer is B. TSH: 0.1-0.5 μU/mL for long-term management of low-risk papillary carcinoma after total thyroidectomy. Regular monitoring and adjustments should be done in collaboration with the treating physician to ensure optimal thyroid hormone replacement and TSH control.	A 60-year-old woman with an 18 mm PTC (low risk), excellent response after 1 year (Tg <0.2 ng/mL, normal ultrasound). What is the TSH target?<br><br>➡ Answer: C. TSH: 0.5-2.0 μU/mL<br>• For excellent response and low risk, TSH can be maintained at normal levels (0.5–2.0 μU/mL).<br>• No need for aggressive TSH suppression.	C. TSH: 0.5-2.0 μU/mL<br>o Explanation: For patients with an excellent response to treatment and low-risk disease, a less stringent TSH suppression goal is appropriate.	**TSH goal after excellent response:**<br>**Answer:** C. TSH: 0.5-2.0 μU/mL.<br>For patients with an excellent response, maintaining TSH in the normal range is appropriate.	Answer: B. TSH: 0.1 - 0.5 μU/mL<br>• For a patient with an excellent response to treatment (undetectable Tg and no structural disease), a mildly suppressed TSH target (0.1-0.5 μU/mL) is appropriate to balance recurrence risk and long-term side effects of TSH suppression.
**9. A 60-year-old woman has undergone total thyroidectomy for classic 18mm papillary carcinoma and has 3 microscopically infiltrated central compartment LNs (1-2mm) without vascular infiltration or microscopic extrathyroidal extension (low to intermediate risk). Would you administer postoperative RAIs?**<br><br>**A. it is very likely**<br>**B. likely**<br>**C. less likely**<br>**D. Unlikely**	Given the low to intermediate risk classification due to the presence of central compartment microscopic lymph node metastases, without vascular infiltration or microscopic extrathyroidal extension, the decision to administer postoperative Radioactive Iodine (RAI) would lean towards being likely (option B).<br>While not all guidelines recommend RAI for low-risk cases, the presence of microscopic lymph node metastases may push the decision towards considering RAI to eliminate any remaining thyroid tissue or microscopic disease. Therefore, the correct answer is B.	In this scenario, the most likely recommendation is: A. It is very likely that the doctor would administer radioactive iodine (RAI) postoperatively.<br>Here's why:<br>Microscopic Lymph Node Metastasis: While the overall risk profile might be considered low to intermediate, the presence of microscopic lymph node metastasis (3 central lymph nodes, 1-2 mm) increases the chance of recurrence. RAI ablation can target these microscopic cancer cells that might not be completely removed by surgery, potentially reducing the risk of recurrence.<br>Low to Intermediate Risk: Although the patient has some risk factors (tumor size and lymph node involvement), other factors like classic variant (generally less aggressive) and no vascular infiltration or extrathyroidal extension suggest a lower overall risk. This makes RAI ablation a more likely recommendation compared to high-risk scenarios.<br>Here's a breakdown of the other options:<br>B. It is likely: This is a reasonable possibility, considering the lymph node involvement.<br>C. It is less likely: While there might be some discussion about the necessity of RAI ablation depending on other factors, the presence of lymph node metastasis makes it less likely that RAI would be ruled out entirely.<br>D. It is unlikely: This is the least likely option due to the presence of lymph node involvement, which is a clear indication for considering RAI ablation in most cases.<br>Important Note:<br>The final decision on RAI ablation should be made by the patient's doctor after considering all the details of the case, including the patient's age, overall health, and the specific findings from the surgery (e.g., microscopic examination of the removed tissue).	Given the patient's low to intermediate risk profile (18 mm classic papillary carcinoma with central compartment microscopic lymph node metastasis without vascular infiltration or microscopic extrathyroidal extension), the decision regarding Radioactive Iodine (RAI) postoperatively is an important consideration. Let's explore the evidence:<br>1. RAI Benefit in Low- to Intermediate-Risk PTC: The use of RAI after total thyroidectomy (TT) remains controversial for low- to intermediate-risk papillary thyroid cancer (PTC) patients. Recent research suggests that RAI may benefit specific subgroups within this risk category.<br>2. Study Findings: A study analyzed data from the Surveillance, Epidemiology, and End Results (SEER) database (2004–2018). Patients were divided into TT (total thyroidectomy) and TT+RAI groups. The following characteristics were more likely to present in the TT+RAI group: multifocality, capsular extension, T3, N1b, and more metastatic cervical lymph nodes. RAI was associated with better overall survival (OS) in low- to intermediate-risk PTC patients in the multivariate Cox regression model. Subgroup analysis showed that RAI predicted better OS in patients ≥55 years, American Joint Committee on Cancer (AJCC) stage II, and capsular extension. However, RAI failed to improve the prognoses of patients with age <55 years, AJCC stage I, PTC ≤1 cm, and capsular invasion.<br>3. Conclusion: RAI was associated with better OS in low- to intermediate-risk PTC patients with specific characteristics (age ≥55 years, multifocality, extrathyroidal extension, and ATA intermediate risk). However, the survival benefit from RAI may be limited in patients with AJCC stage I, PTC ≤1 cm, unifocality, capsular invasion, and ATA low-risk diseases, even if they have pathological cervical lymph node metastasis.<br>Therefore, based on the available evidence, the correct answer is D. It is unlikely that RAI would be administered postoperatively for this patient. Always consult with an endocrinologist or thyroid specialist to tailor the management plan based on individual factors and preferences.	A 60-year-old woman with an 18 mm PTC and 3 microscopic central LN metastases (low to intermediate risk). Would you give RAI ablation?<br><br>➡ Answer: B. Likely<br>• ATA suggests RAI for intermediate-risk patients, particularly those with multiple microscopic LN metastases.	B. likely<br>o Explanation: Microscopic central compartment lymph node involvement increases the risk of recurrence. Therefore RAI is more likely to be used in this situation.	**Post-total thyroidectomy with microscopic LN involvement (low to intermediate risk):**<br>**Answer:** B. Likely.<br>RAI may be considered for intermediate-risk cases with microscopic lymph node involvement.	Answer: B. likely<br>• For low to intermediate risk (e.g., microscopic lymph node involvement), postoperative RAI is often recommended to reduce the risk of recurrence.

	TIME 1			TIME 2			
	**ChatGPT**	**Gemini**	**Copilot**	**ChatGPT**	**Gemini**	**Copilot**	**DeepSeek**
**10. A 60-year-old woman has undergone total thyroidectomy for classic papillary carcinoma of 18 mm and has no known infiltrated LN or no vascular infiltration while she has microscopic extrathyroidal extension (low to intermediate risk). Would you administer postoperative RAIs?**<br><br>**A. it is very likely**<br>**B. likely**<br>**C. less likely**<br>**D. Unlikely**	Given the presence of microscopic extrathyroidal extension in addition to the absence of lymph node metastasis or vascular infiltration, the case is classified as low to intermediate risk. The decision to administer postoperative Radioactive Iodine (RAI) would lean towards being likely (option B).<br>Microscopic extrathyroidal extension increases the risk of recurrence and may warrant RAI therapy to eliminate any remaining thyroid tissue or microscopic disease. However, the decision should be made based on a comprehensive assessment of the patient's individual risk factors and discussion between the patient and their healthcare provider.<br>Therefore, the correct answer is B.	In this scenario, the most likely recommendation is: B. It is likely that the doctor would administer radioactive iodine (RAI) postoperatively.<br>Here's why:<br>Microscopic Extrathyroidal Extension (ETE): While the overall risk profile might be considered low to intermediate, the presence of microscopic ETE indicates that some cancer cells have spread beyond the thyroid capsule. RAI ablation can be beneficial in destroying these microscopic deposits, potentially reducing the risk of recurrence.<br>Other Factors: The absence of lymph node metastasis and vascular infiltration are positive factors suggesting a lower overall risk. However, the presence of ETE outweighs these aspects in this case.<br>Low to Intermediate Risk: The patient has a mix of risk factors, making RAI ablation a more likely recommendation compared to strictly low-risk scenarios.<br>Here's a breakdown of the other options:<br>A. It is very likely: This is a possibility, but the lack of lymph node involvement suggests a slightly lower likelihood compared to cases with both ETE and lymph node metastasis.<br>C. It is less likely: While there might be some discussion depending on the specific details, the presence of ETE makes it less likely that RAI would be entirely ruled out.<br>D. It is unlikely: This is the least likely option due to the ETE, which is a clear indication for considering RAI ablation in most cases.<br>Important Note:<br>The final decision on RAI ablation should be made by the patient's doctor after considering all the details of the case, including the patient's age, overall health, the extent of microscopic ETE, and the specific findings from the surgery (e.g., microscopic examination of the removed tissue).	Given the patient's low to intermediate risk profile (18 mm classic papillary carcinoma with central compartment microscopic lymph node metastasis without vascular infiltration or microscopic extrathyroidal extension), the decision regarding Radioactive Iodine (RAI) postoperatively is an important consideration. Recent research sheds light on this topic. Let's explore the evidence:<br>1. Study Findings: A study analyzed data from the Surveillance, Epidemiology, and End Results (SEER) database (2004–2018). Patients were divided into two groups: those who underwent total thyroidectomy (TT) alone and those who received TT+RAI. The following characteristics were more likely to present in the TT+RAI group: multifocality, capsular extension, T3, N1b, and more metastatic cervical lymph nodes. RAI was associated with better overall survival (OS) in low- to intermediate-risk papillary thyroid cancer (PTC) patients in the multivariate Cox regression model. Subgroup analysis showed that RAI predicted better OS in patients ≥55 years, American Joint Committee on Cancer (AJCC) stage II, and capsular extension. However, RAI failed to improve the prognoses of patients with age <55 years, AJCC stage I, PTC ≤1 cm, and capsular invasion.<br>2. Conclusion: RAI was associated with better OS in low- to intermediate-risk PTC patients with specific characteristics (age ≥55 years, multifocality, extrathyroidal extension, and ATA intermediate risk). However, the survival benefit from RAI may be limited in patients with AJCC stage I, PTC ≤1 cm, unifocality, capsular invasion, and ATA low-risk diseases, even if they have pathological cervical lymph node metastasis.<br>Therefore, based on the available evidence, the correct answer is D. It is unlikely that RAI would be administered postoperatively for this patient. Always consult with an endocrinologist or thyroid specialist to tailor the management plan based on individual factors and preferences.	A 60-year-old woman with an 18 mm PTC, no LN involvement, no vascular invasion, but microscopic extrathyroidal extension (low to intermediate risk). Would you give RAI ablation?<br><br>➡ Answer: C. Less likely<br>• Microscopic extrathyroidal extension alone is not a strong indication for RAI.<br>• RAI may be considered, but not routinely recommended.	B. likely<br>o Explanation: Microscopic extrathyroidal extension increases the risk of recurrence and therefore RAI is more likely to be used in this case.	**Post-total thyroidectomy with microscopic extrathyroidal extension (low to intermediate risk):**<br>**Answer:** B. Likely.<br>RAI is often recommended for intermediate-risk cases with microscopic extrathyroidal extension.	Answer: B. likely<br>• Microscopic extrathyroidal extension places the patient in the low to intermediate risk category, making postoperative RAI likely to be recommended.
**11. To the woman of question 9 and 10, if you decided to administer RAI postoperatively, what would be the dose?**<br><br>**A. 30mCi**<br>**B. 50mCi**<br>**C. 70mCi**<br>**D. 100mCi**	In patients with low to intermediate risk papillary carcinoma who undergo total thyroidectomy and are candidates for postoperative Radioactive Iodine (RAI) therapy, the recommended dose is typically higher compared to those with low-risk tumors.<br>For patients with low to intermediate risk, a commonly recommended RAI dose is around 50 to 100 mCi (millicurie) depending on various factors including tumor size, extent of disease, and individual patient characteristics.<br>Therefore, the correct answer would be:<br>D. 100 mCi	Similar to the previous case, I cannot recommend a specific radioactive iodine (RAI) dose (A, B, C, or D) for this patient. The appropriate RAI dosage depends on several factors and should be determined by the patient's doctor. Here's why:<br>Individualized Treatment: RAI ablation dosage varies depending on the patient's specific risk profile, including factors like:<br>Age<br>Overall health<br>Tumor size (18 mm in this case)<br>Extent of microscopic extrathyroidal extension<br>Thyroid stimulating hormone (TSH) levels<br>Patient's preferences<br>Risk Stratification: Doctors often use risk stratification tools to determine the appropriate RAI dose. These tools consider various factors and categorize the patient's risk of recurrence (low, intermediate, high). The dose is then adjusted based on the risk category. Microscopic ETE elevates the risk profile compared to a case without ETE.<br>Diagnostic Scan vs. Ablation Dose: There might be a preliminary low-dose RAI scan before the actual ablation treatment to assess thyroid tissue remnants or microscopic cancer spread. The ablation dose itself would be higher.<br>Recommendation:<br>The best course of action is for the patient to discuss RAI ablation with their doctor. The doctor will consider all the relevant factors and recommend the most appropriate RAI dose for this specific case. They will likely choose a dose within the range for intermediate-risk patients considering the presence of microscopic ETE.	For patients with low to intermediate risk papillary thyroid carcinoma (PTC) who have undergone total thyroidectomy, the decision regarding Radioactive Iodine (RAI) postoperatively is crucial. Let's consider the available evidence:<br>1. Study Findings: A study analyzed data from the Surveillance, Epidemiology, and End Results (SEER) database (2004–2018). Patients were divided into two groups: those who underwent total thyroidectomy (TT) alone and those who received TT+RAI. The following characteristics were more likely to present in the TT+RAI group: multifocality, capsular extension, T3, N1b, and more metastatic cervical lymph nodes. RAI was associated with better overall survival (OS) in low- to intermediate-risk PTC patients in the multivariate Cox regression model. Subgroup analysis showed that RAI predicted better OS in patients ≥55 years, American Joint Committee on Cancer (AJCC) stage II, and capsular extension. However, RAI failed to improve the prognoses of patients with age <55 years, AJCC stage I, PTC ≤1 cm, and capsular invasion.<br>2. Conclusion: RAI was associated with better OS in low- to intermediate-risk PTC patients with specific characteristics (age ≥55 years, multifocality, extrathyroidal extension, and ATA intermediate risk). However, the survival benefit from RAI may be limited in patients with AJCC stage I, PTC ≤1 cm, unifocality, capsular invasion, and ATA low-risk diseases, even if they have pathological cervical lymph node metastasis. Considering this evidence, let's recommend an appropriate RAI dose: For patients with low to intermediate risk, a lower RAI dose is typically recommended.<br>Based on the available information, I suggest considering a dose of 50 mCi for postoperative RAI treatment in these patients. However, individualized decisions should be made in consultation with an endocrinologist or thyroid specialist, considering the patient's unique characteristics and preferences. Therefore, the correct answer is B. 50 mCi. Always consult with the treating physician to tailor the management plan based on individual factors and clinical judgment.	For patients in Questions 9 and 10, if RAI was given, what dose would you use?<br><br>➡ Answer: A. 30 mCi<br>• Low-dose (30 mCi) RAI is sufficient for intermediate-risk patients with microscopic LN metastases or minimal extrathyroidal extension.	D. 100 mCi<br>o Explanation: When RAI is needed for intermediate risk patients, a higher dose is used.	**RAI dose for intermediate-risk cases:**<br>**Answer:** B. 50 mCi.<br>A moderate dose (50 mCi) is typically used for intermediate-risk cases.	Answer: B. 50 mCi<br>• For low to intermediate risk patients, a moderate dose of RAI (50 mCi) is typically used.
**12. A 60-year-old woman has undergone total thyroidectomy for classic 18mm papillary carcinoma and has 3 microscopically infiltrated central compartment LNs (1-2mm) without vascular infiltration or extrathyroidal extension (low to intermediate risk). After one year the baseline levels of Tg are <0.2ng/mL (with negative anti-Tg) and cervical ultrasound without findings (excellent response). What is the goal of TSH:**<br><br>**A. TSH<0.1μU/mL**<br>**B. TSH:0.1-0.5μU/mL**<br>**C. TSH:0.5-2.0 μU/mL**	Given the excellent response to treatment with baseline thyroglobulin levels <0.2 ng/mL and negative anti-thyroglobulin antibodies, the goal of thyroid-stimulating hormone (TSH) suppression would be to maintain it within the low-normal range to minimize the risk of recurrence while avoiding the potential adverse effects of over-suppression.<br>Therefore, the correct answer is:<br>C. TSH: 0.5-2.0 μU/mL	The most likely TSH goal in this case is: B. TSH: 0.1-0.5 μU/mL.<br>Here's why:<br>The American Thyroid Association (ATA) guidelines recommend different TSH targets for papillary thyroid cancer patients depending on their risk of recurrence.<br>This case presents with an excellent response:<br>Low baseline thyroglobulin (<0.2ng/mL) with negative anti-thyroglobulin antibodies suggests minimal to no detectable thyroid tissue.<br>No evidence of recurrence on cervical ultrasound.<br>Given this low to intermediate risk profile with an excellent response, the TSH goal would likely fall within the 0.1-0.5 μU/mL range. This level mildly suppresses TSH, potentially reducing the risk of cancer recurrence while minimizing the potential side effects of overly suppressed TSH.<br>Here's a breakdown of the other options:<br>A. TSH < 0.1μU/mL (strict suppression): This might be considered for high-risk patients, but with an excellent response and a lower overall risk profile due to the lack of ETE, stricter suppression (TSH < 0.1 μU/mL) might not be necessary.<br>C. TSH: 0.5-2.0 μU/mL (normal range): This could be an option for even lower risk scenarios or after a longer period of successful follow-up with excellent response. However, with microscopic lymph node involvement, maintaining a mildly suppressed TSH level might be preferred for a while.<br>Important Note:<br>The final decision on the ideal TSH target should be made by the patient's doctor after considering all the details of the case, including long-term follow-up results and the patient's individual health status.	For a patient who has undergone total thyroidectomy for an 18 mm classic papillary carcinoma with central compartment microscopic lymph node metastasis (1-2 mm) but without vascular infiltration or microscopic extrathyroidal extension, the goal of TSH (Thyroid Stimulating Hormone) management is crucial for long- term follow-up.<br>Let's consider the available evidence and guidelines:<br>1. Risk Assessment: The patient's tumor characteristics fall into the low to intermediate risk category. The absence of adverse features (such as vascular infiltration or extrathyroidal extension) indicates a favorable prognosis.<br>2. TSH Suppression Guidelines: The American Thyroid Association (ATA) provides recommendations for TSH suppression based on risk stratification:<br>High-Risk and Intermediate-Risk Patients: Achieve an initial TSH level below 0.1 mU/L. Low-Risk Patients: Maintain TSH at or slightly below the lower limit of normal, which corresponds to a range of 0.1–0.5 mU/L.<br>3. Long-Term Management: After the initial phase, the goal is to maintain TSH within an appropriate range. For low-risk to intermediate-risk papillary thyroid carcinoma (PTC) patients, the ATA recommends a TSH level between 0.5 and 2.0 mU/L, which falls within the normal range.<br>Therefore, the correct answer is C. TSH: 0.5-2.0 μU/mL for long-term management of this patient. Regular monitoring and adjustments should be done in collaboration with the treating physician to ensure optimal thyroid hormone replacement and TSH control.	A 60-year-old woman with 3 microscopic central LN metastases (low to intermediate risk), excellent response (Tg <0.2 ng/mL, negative ultrasound) after 1 year. What is the TSH target?<br><br>➡ Answer: B. TSH: 0.1-0.5 μU/mL<br>• TSH suppression (0.1–0.5 μU/mL) is recommended for intermediate-risk patients, even with an excellent response.<br>• More aggressive suppression (TSH <0.1) is not needed due to the low tumor burden.	B. TSH: 0.1-0,5μU/mL<br>o Explanation: Patients with intermediate-risk disease often require more stringent TSH suppression to minimize the risk of recurrence.	**TSH goal after excellent response in intermediate-risk cases:**<br>**Answer:** B. TSH: 0.1-0.5 μU/mL.<br>Mild TSH suppression is recommended for intermediate-risk cases with an excellent response.	Answer: A. TSH < 0.1 μU/mL<br>• For patients with low to intermediate risk and an excellent response, TSH suppression to <0.1 μU/mL is recommended to minimize the risk of recurrence.
